# Eukaryotic initiation factor 5B (eIF5B) provides a critical cell survival switch to glioblastoma cells via regulation of apoptosis

**DOI:** 10.1038/s41419-018-1283-5

**Published:** 2019-01-22

**Authors:** Joseph A. Ross, Keiran Vanden Dungen, Kamiko R. Bressler, Mikayla Fredriksen, Divya Khandige Sharma, Nirujah Balasingam, Nehal Thakor

**Affiliations:** 1 Department of Chemistry and Biochemistry, University of Lethbridge, 4401 University Drive W, Lethbridge, AB T1K 3M4 Canada; 2 Canadian Centre for Behavioral Neuroscience (CCBN), Department of Neuroscience, University of Lethbridge, 4401 University Drive W, Lethbridge, AB T1K 3M4 Canada; 3 Arnie Charbonneau Cancer Institute, Cumming School of Medicine, University of Calgary, 3280 Hospital Drive NW, Calgary, AB T2N 4Z6 Canada

## Abstract

Physiological stress conditions attenuate global mRNA translation via modifications of key eukaryotic initiation factors. However, non-canonical translation initiation mechanisms allow cap-independent translation of certain mRNAs. We have previously demonstrated that eIF5B promotes cap-independent translation of the mRNA encoding the antiapoptotic factor, XIAP, during cellular stress. Here, we show that depletion of eIF5B sensitizes glioblastoma multiforme cells to TRAIL-induced apoptosis by a pathway involving caspases-8, −9, and −7, with no significant effect on cell cycle progression. eIF5B promotes evasion of apoptosis by promoting the translation of several IRES-containing mRNAs, encoding the antiapoptotic proteins XIAP, Bcl-xL, cIAP1, and c-FLIP_S_. We also show that eIF5B promotes translation of nuclear factor erythroid 2-related factor 2 and suggest that reactive oxygen species contribute to increased apoptosis under conditions of eIF5B depletion. Finally, eIF5B depletion leads to decreased activation of the canonical NF-κB pathway. Taken together, our data suggest that eIF5B represents a regulatory node, allowing cancer cells to evade apoptosis by promoting the translation of pro-survival proteins from IRES-containing mRNAs.

## Introduction

Eukaryotic translation exists in two primary forms: canonical, which makes use of an m^7^G cap structure at the 5’ end of the mRNA, and non-canonical, which relies on alternative means of ribosome recruitment, such as internal ribosome entry sites (IRESs)^1^. Physiological stress conditions attenuate global mRNA translation owing to modifications of key eukaryotic initiation factors. For example, phosphorylation of eIF2α inhibits its ability to deliver met-tRNAi to the 40 S ribosome, preventing translation initiation. However, non-canonical translation initiation mechanisms allow for selective translation of certain mRNAs under such conditions. These mRNAs often encode stress–response proteins and dysregulation of non-canonical translation initiation is implicated in disease states like cancer^1,2^. Although IRESs were originally discovered in viruses, they have since been shown to exist in a variety of eukaryotic mRNAs^3–5^. For instance, nuclear factor erythroid 2-related factor 2 (Nrf2) can be translated from an IRES under conditions of eIF2α phosphorylation^6^. Similarly, several antiapoptotic proteins can be translated from IRESs, such as X-linked inhibitor of apoptosis (XIAP)^7^, cellular inhibitor of apoptosis protein 1 (cIAP1)^8^, and B-cell lymphoma extra-large (Bcl-xL)^9^. The short isoform of cellular FLICE-like inhibitory protein (c-FLIP_S_) also encodes a putative IRES^4^. These proteins play critical roles in regulating both intrinsic and extrinsic apoptotic pathways^10–13^.

Under conditions of cellular stress and eIF2α phosphorylation, IRES-dependent translation of XIAP mRNA relies on eIF5B^7^. eIF5B is homologous to bacterial and archaeal IF2, which delivers met-tRNA^fMet^ to bacterial/archaeal ribosomes^14^. Under standard conditions, eIF5B is responsible for assisting in the joining of the 40 S and 60 S ribosomal subunits, as well as playing a role in stabilizing met-tRNAi binding^15^. eIF5B was also shown to deliver met-tRNAi into the P-site of the ribosome in an IRES-dependent translation initiation mechanism utilized by the CSFV (classical swine fever virus) and HCV (Hepatitis C virus) IRESs^16–18^. Thus, eIF5B is capable of substituting for eIF2 in met-tRNAi-delivery to the ribosome. Recently, eIF5B was shown to act as an essential factor for cap-dependent translation of hypoxia-response proteins in hypoxic glioblastoma (GBM) cells^19^. eIF5B has also been shown to regulate cell cycle progression via regulating upstream open reading frame-containing mRNAs, such as p27 and p21^20^. These findings suggest a non-canonical role for eIF5B under cellular stress conditions. Moreover, levels of eIF5B are elevated in several malignancies and eIF5B can be classified as an oncogenic stress-related protein.

However, a precise role of eIF5B in cancer progression has not been defined. We thus sought to determine whether eIF5B has a role in the viability of cancer cells. To this end, we primarily used U343 (GBM cells) as a model. In this study, we report that siRNA-mediated depletion of eIF5B increased the sensitivity of GBM cells, but not immortalized fibroblasts, to TRAIL-induced apoptosis. We show that eIF5B depletion synergizes with TRAIL to activate apoptosis by a pathway involving caspases-8, −9, and −7. We demonstrate that eIF5B promotes evasion of apoptosis by a mechanism involving the translational upregulation of several IRES-containing mRNAs of antiapoptotic proteins, including XIAP, Bcl-xL, cIAP1, and c-FLIP_S_. We also show that eIF5B promotes translation of p21 without affecting cell cycle progression. We demonstrate that eIF5B promotes translation of Nrf2 and suggest that ROS contribute to increased apoptosis under conditions of eIF5B depletion. Finally, we show that eIF5B-silencing leads to decreased activation of the canonical NF-κB pathway. This is the first demonstration that eIF5B regulates the translation of such a wide variety of apoptosis-related proteins. Taken together, our data suggest that eIF5B represents a regulatory node that promotes translation of mRNAs encoding pro-survival proteins, thus allowing GBM cells to evade apoptosis.

## Results

### eIF5B promotes resistance to apoptosis-inducing agents

**Fig. 1:**
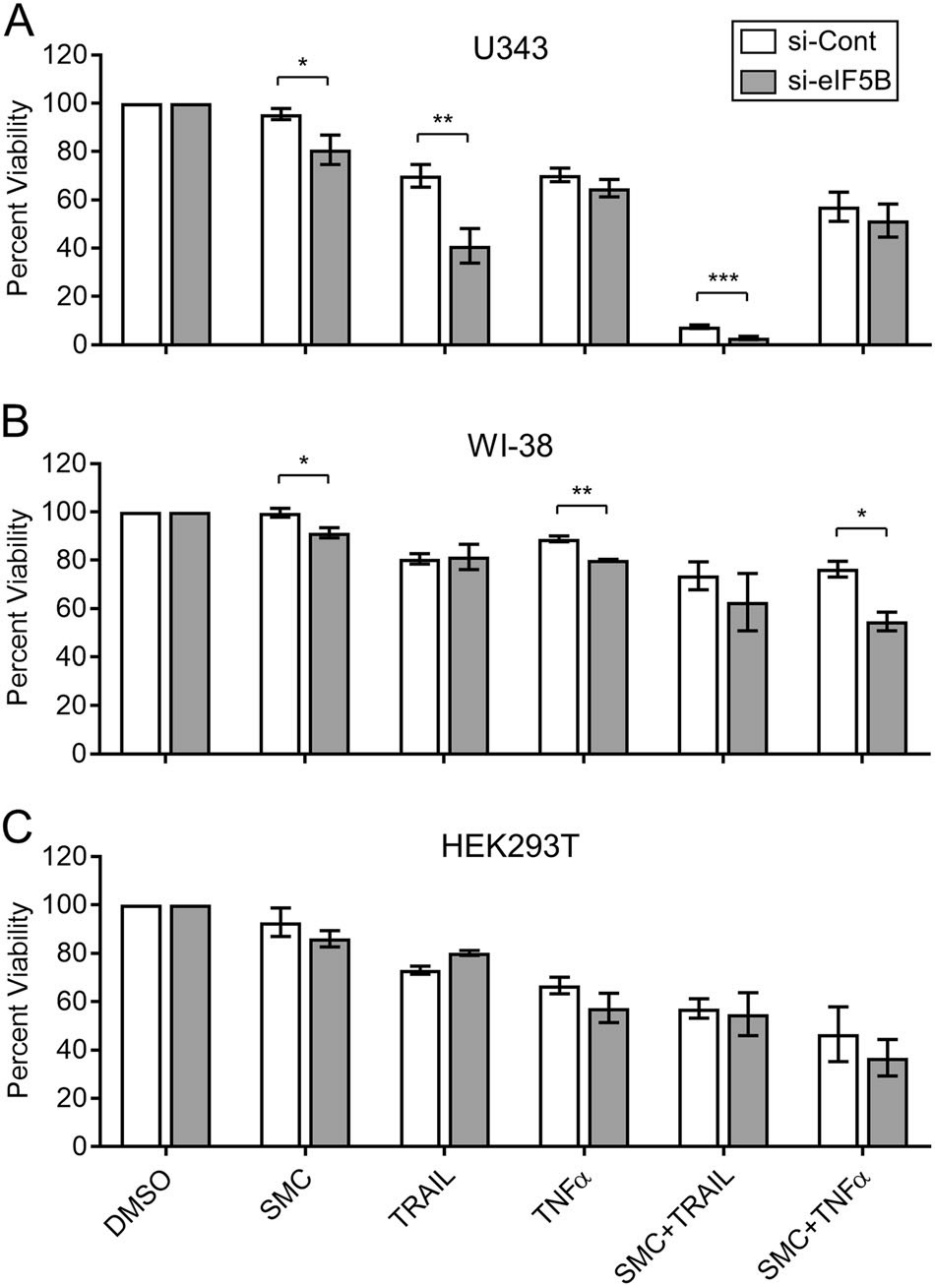
Depletion of eIF5B sensitizes U343 glioblastoma cells, but not WI-38 and HEK293T cells, to TRAIL. U343 cells **a**, WI-38 cells **b**, or HEK293T cells **c** were transfected with a non-specific control siRNA (si-Cont) or an eIF5B-specific siRNA pool (si-eIF5B). After 24 h, the cells were treated with a vehicle control (0.5% DMSO), TL-32711 (SMC; 10 µm), TRAIL (100 ng/mL), or TNF-α (100 ng/mL). Cell viability was measured after a further 72 h. The resulting fluorescence readings are expressed as percent viability, where the readings for control or eIF5B-depleted cells were each normalized to the vehicle treatment. Data are expressed as mean ± SEM for three (WI-38, HEK293T) or six (U343) independent biological replicates. *, *p* < 0.05; **, *p* < 0.01; ***, *p* < 0.001

To test whether eIF5B promotes GBM cell viability, we used RNA interference to deplete eIF5B in five established GBM cell lines (U343, U251N, A172, U373, and U87MG) with diverse genetic backgrounds (p53, PTEN, EGFR, and MGMT status) (Table S1). Using a pool of three eIF5B-specific siRNAs, we were able to achieve a reduction of ~ 90% in eIF5B protein levels relative to cells treated with a non-specific control siRNA (Figure S1A). This was also the case for two immortalized but non-cancerous cells lines, human embryonic kidney cells (HEK293T) and lung fibroblasts (WI-38) (Figure S1A). We used the alamarBlue assay^21^ to screen for any effects on cell proliferation or viability. Silencing of eIF5B alone caused no significant decrease in viability for all cell lines tested (Figure S1B). We next tested whether silencing eIF5B would sensitize GBM cells to pro-apoptotic compounds. Depletion of eIF5B led to a robust sensitization of U343 cells to TRAIL (a pro-death cytokine that activates receptor-mediated apoptosis) but had no significant effect on sensitivity to another cytokine, tumor necrosis factor-α (TNF-α) (Fig. 1a). Interestingly, TNF-α enhanced c-FLIP_S_ levels in both eIF5B-depleted and control cells (Figure S2A), possibly explaining why eIF5B-depletion did not enhance cell death in TNF-α-treated cells. Smac mimetic compounds (SMCs) have been used to inhibit inhibitors of apoptosis (IAPs) in combination with other pro-death stimuli, such as TRAIL^22^. Therefore, we tested the effect of eIF5B depletion on the sensitivity of U343 cells to the SMC, TL-32711^23^. Depletion of eIF5B caused a modest sensitization to TL-32711 (Fig. 1a). eIF5B depletion also caused a significant sensitization to the combination of SMC + TRAIL, which suggests that eIF5B silencing sensitizes cells to TRAIL by a mechanism that is at least partially synergistic with SMC (Fig. 1a).

Similar experiments were conducted in the other GBM lines (U251N, U373, A172, and U87MG), with similar trends in U251N (Figure S1C). Interestingly, of the GBM cell lines tested, only U343, U251N, and A172 displayed decreased phosphorylation of EGFR upon eIF5B depletion (Figure S1D), which would inhibit EGFR-mediated pro-growth pathways. A172 was already TRAIL-sensitive, masking any potential eIF5B-dependent phenotypes (Figure S1C). None of the other genotypes (e.g., p53 status; Table S1) correlated with the phenotype. TRAIL is a potential cancer therapy that selectively kills cancer cells while non-malignant cells remain viable^24^. We chose two TRAIL-resistant cell lines (WI-38 and HEK293T) to ensure that eIF5B depletion does not sensitize non-cancerous cells to this potential therapeutic agent. Figure 1 shows that, overall, both WI-38 and HEK293T cells respond similarly to the various compounds tested. However, although both cell lines respond to TRAIL (~20% decreased viability) at the concentration used here, neither becomes further sensitized by eIF5B-depletion (Fig. 1b, c). This suggests that eIF5B promotes TRAIL resistance specifically in a subset of cancerous cells and not in immortalized somatic cells.

De-convolution of the eIF5B siRNA mix confirmed that TRAIL sensitization was owing to silencing eIF5B and not an off-target effect (Figure S3A, B). The interaction between eIF1A and eIF5B promotes translation^25^, suggesting that a change in eIF5B levels might affect those of eIF1A. However, eIF5B depletion did not affect eIF1A levels (Figure S3C). Silencing of another translation factor, eIF3F, had little effect on U343 cell viability (Figure S3D) and did not sensitize the cells to TRAIL (Figure S3E), suggesting that the phenotype is specific to eIF5B. Taken together, these data indicate that eIF5B depletion has a specific effect on the sensitivity of a subset of GBM cells to TRAIL.

### Depletion of eIF5B enhances TRAIL-induced apoptosis by a caspase-8/9/7-mediated pathway

**Fig. 2:**
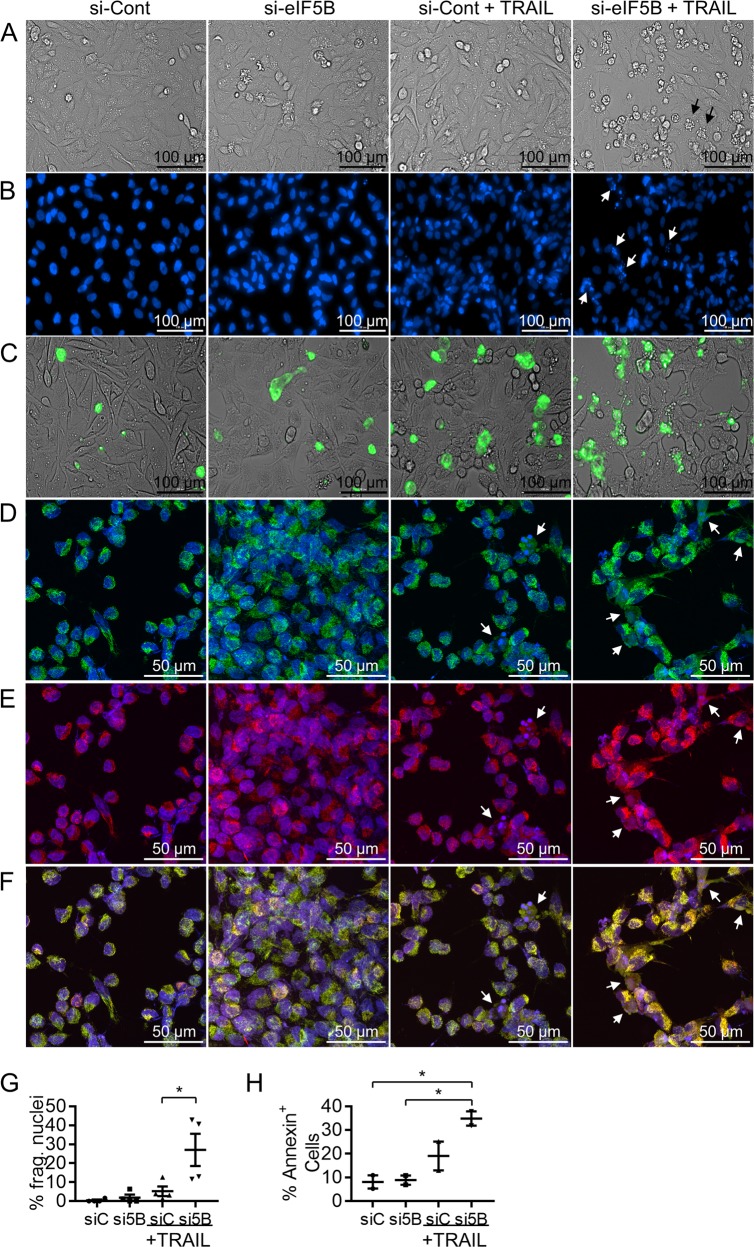
Depletion of eIF5B enhances TRAIL-induced apoptotic cell behavior. U343 cells were treated as in Fig. 1, except that TRAIL treatment was limited to the last 4 h. Cells in **a** were imaged at × 20 magnification by brightfield microscopy. Cells in **b**, **c** were, respectively, imaged by fluorescence microscopy to analyze Hoechst-stained nuclear DNA (blue) or Annexin V-FITC-positive cells (green). Alternatively, the cells in **d–f** were analyzed by confocal microscopy at × 40 magnification, using immunocytochemistry to assess the sub-cellular localization of cytochrome c. **d** Cyt C (green) with a Hoechst DNA counterstain (blue). **e** MitoTracker Red with Hoechst counterstain. **f** Overlay of images from **d**, **e**. The black arrows in **a** indicate examples of cells apparently undergoing apoptosis. The white arrows in **b** indicate examples of nuclear fragmentation. The white arrows in **d**–**f** indicate examples of apparently disrupted mitochondria. The percent of Hoechst-stained nuclei from **b** displaying fragmentation (white arrows) were quantified **g**, as were the percent of annexin-positive cells (green) from **c** **h**. Data are expressed as scatter plots, with horizontal bars representing the mean and error bars indicating the SEM for four **g** or two **h** biological replicates. *, *p* < 0.05

**Fig. 3:**
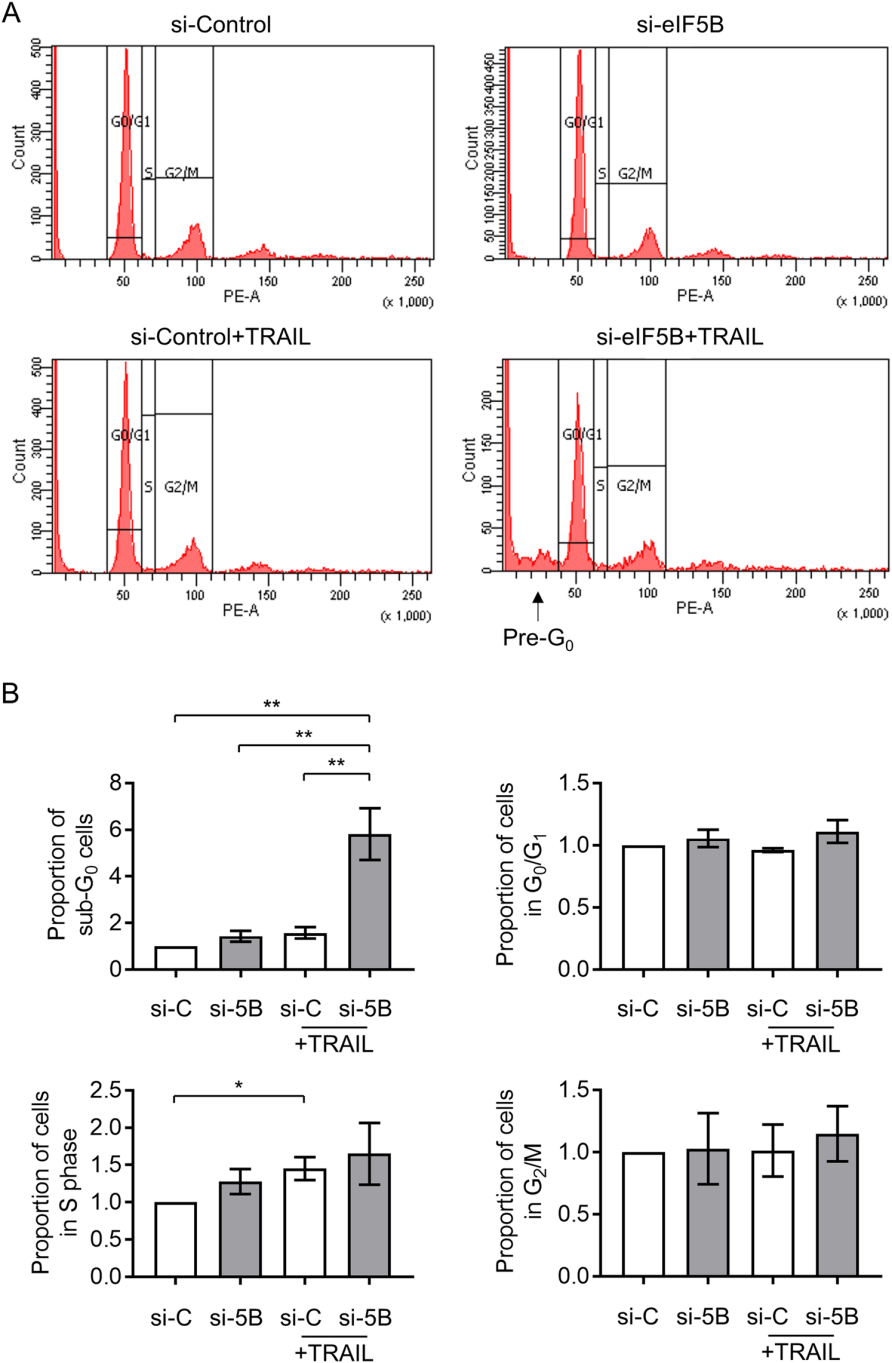
Depletion of eIF5B enhances TRAIL-induced apoptosis without affecting cell cycle progression. U343 cells were treated as in Fig. 1 before analyzing cellular DNA content by propidium iodide staining and flow cytometry. **a** Representative images showing the number of cells in G_0_/G_1_, S, or G_2_/M phases, as well as sub-G_0_ cells. **b** Percentage of sub-G_0_ cells (top left), cells in G_0_/G_1_ (top right), S-phase (bottom left), and G_2_/M (bottom right). siC, non-specific siRNA; si5B, eIF5B-specific siRNA. Data are expressed as mean ± SEM for four independent biological replicates. *, *p* < 0.05; **, *p* < 0.01

To confirm that decreased alamarBlue activity in eIF5B-silenced, TRAIL-treated cells was owing to increased apoptosis, we performed a series of microscopy experiments. First, brightfield microscopy confirmed that TRAIL-treated, eIF5B-depleted U343 cells displayed a classical apoptotic morphology, including membrane blebbing and the expulsion of apoptotic bodies (Fig. 2a). Hoechst live-cell nuclear staining revealed increased nuclear fragmentation in TRAIL-treated, eIF5B-depleted cells (Fig. 2b, g), and a greater proportion of these cells stained positive for Annexin V-FITC, relative to untreated cells (Fig. 2c, h). Annexin V binds phosphatidylserine, which is a marker of apoptosis when located on the outer leaflet of the plasma membrane^26^. We next investigated whether eIF5B depletion enhanced the release of cytochrome c from mitochondria in TRAIL-treated cells. Immunofluorescence microscopy with a cyt c-specific antibody demonstrated that cyt c localization (green) was punctate, well-separated from the nuclei (Hoechst counterstain; blue), and overlaid well with a mitochondrial stain (MitoTracker Red) in untreated control cells (Fig. 2d–f). However, eIF5B depletion plus TRAIL treatment led to a more diffuse cyt c distribution (Fig. 2d–f, left to right). The distribution of MitoTracker Red became similarly diffuse, suggesting that eIF5B depletion plus TRAIL led to a general disruption of mitochondria. In an orthogonal assay, we fractionated mitochondrial versus cytosolic proteins and immunoblotted for cyt c. Silencing eIF5B did not lead to cyt c release from mitochondria in either TRAIL-treated or untreated cells (Figure S4). Moreover, propidium iodide staining and flow cytometry demonstrated that, relative to untreated control cells, eIF5B depletion and/or TRAIL treatment had no significant effect on the percentage of U343 cells in G_0_/G_1_, S, or G_2_/M phases of the cell cycle (Fig. 3), ruling out an effect of eIF5B or TRAIL on cell cycle progression. However, TRAIL-treated, eIF5B-silenced cells showed a significant increase in pre-G_0_ peaks (i.e., more cells had less than a full complement of DNA), further suggesting that the viability phenotype is owing to increased apoptosis (Fig. 3).

**Fig. 4:**
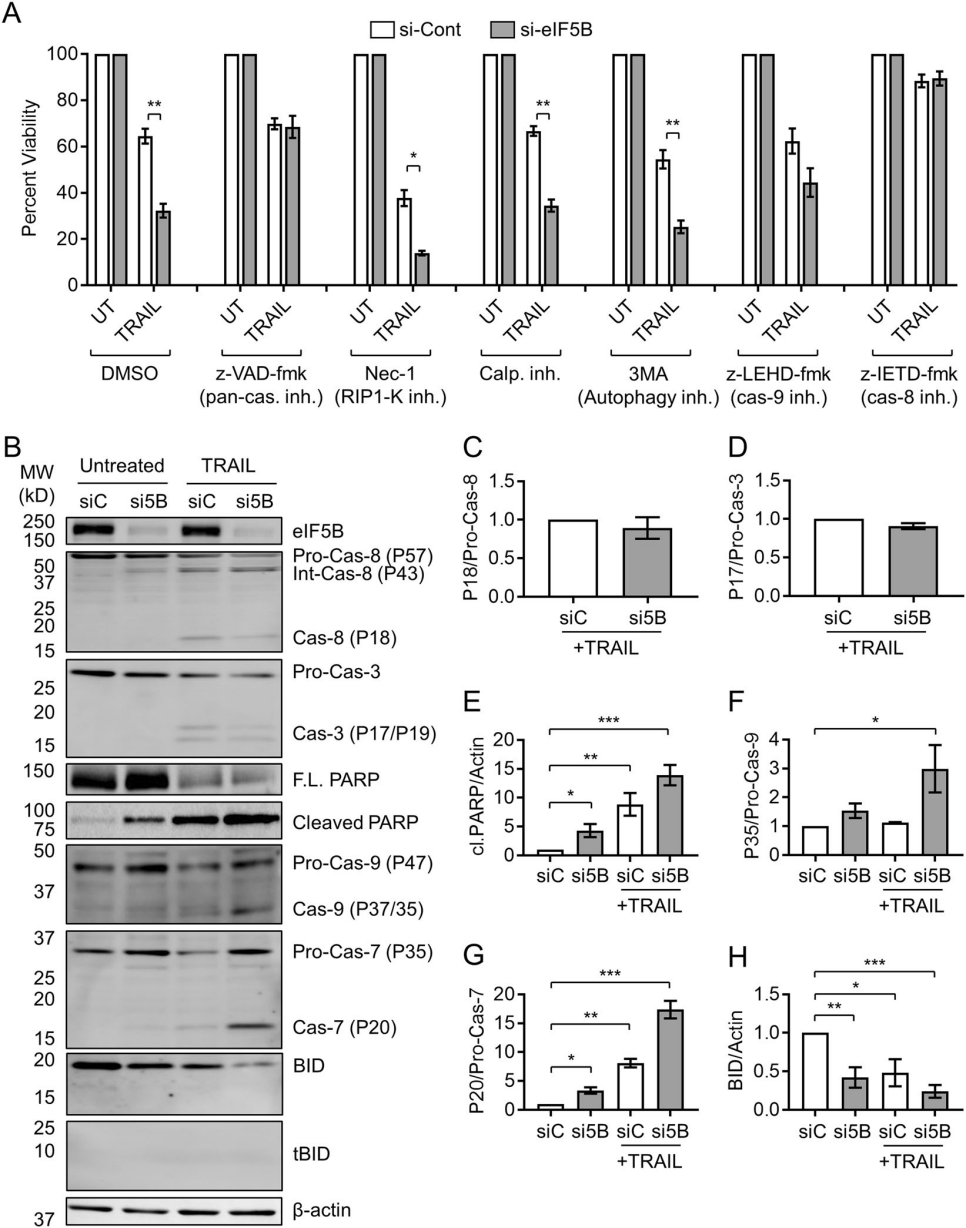
Depletion of eIF5B enhances TRAIL-induced apoptosis by a caspase-8/9/7-mediated pathway. **a** Control or eIF5B-depleted cells were pre-treated for 2 h with a vehicle control (0.5% DMSO), pan-caspase inhibitor (z-VAD-fmk; 20 µm), RIP1-K inhibitor (Nec-1; 100 µm), calpain inhibitor III (Calp. inh.; 100 nm), autophagy inhibitor (3-Methyladenine; 3MA; 5 mm), caspase-9 inhibitor (z-LEHD-fmk; 50 µm), or caspase-8 inhibitor (z-IETD-fmk; 50 µm), before being treated with TRAIL as in Fig. 1. **b**–**h** Control or eIF5B-depleted cells (without caspase inhibition) were treated 4 h in the presence or absence of 100 ng/mL TRAIL, harvested in RIPA lysis buffer, and 20 µg of total protein resolved by SDS-PAGE before performing immunoblotting. **b** Representative images of immunoblots probing for eIF5B, caspase-8, caspase-3, full-length (F.L.) PARP, cleaved PARP, caspase-9, caspase-7, total Bid, tBid, and β-actin (internal control). **c–h** Quantitation of active- versus pro-caspase-8 **c**, active- versus pro-caspase-3 **d**, cleaved PARP normalized to β-actin **e**, active- versus pro-caspase-9 **f**, active- versus pro-caspase-7 **g**, and total Bid normalized to β-actin **h**. siC, non-specific siRNA; si5B, eIF5B-specific siRNA. Data are expressed as mean ± SEM for three independent biological replicates. *, *p* < 0.05; **, *p* < 0.01; ***, *p* < 0.001

Next, we assessed whether cell death in TRAIL-treated, eIF5B-depleted cells might also be owing to alternative mechanisms like necroptosis or autophagy, as both are known to result from TRAIL treatment^27,28^. We assessed the effect of eIF5B-depletion on TRAIL-induced death in an alamarBlue assay by pre-treating U343 cells with a pan-caspase inhibitor (z-VAD-fmk), a RIP1-kinase inhibitor (Necrostatin-1), an autophagy inhibitor (3-methyladenine; 3MA), or calpain inhibitor III. Relative to a vehicle control, z-VAD-fmk prevented the effects of eIF5B depletion on TRAIL sensitivity, confirming that apoptosis is essential to the eIF5B-depletion/TRAIL viability phenotype (Fig. 4a). Conversely, the phenotype was not reversed by Necrostatin-1, calpain inhibitor III, or 3MA, indicating that eIF5B does not affect the ability of TRAIL to induce necroptosis, calpain-dependent cell death, or autophagy (Fig. 4a).

Inhibitors specific to caspases-8 or -9 (z-IETD-fmk or z-LEHD-fmk, respectively) abrogated the eIF5B-depletion/TRAIL phenotype (Fig. 4a), suggesting that eIF5B silencing sensitizes cells to a caspases-8/9-dependent pathway. Western blot analysis revealed that TRAIL activated caspases-8 and −3 (as expected) but eIF5B depletion caused no further activation (Fig. 4b, c, d). However, PARP was further cleaved in TRAIL-treated, eIF5B-depleted cells (Fig. 4b, e). PARP is cleaved by active caspases-3 and −7^29^. TRAIL treatment and eIF5B-depletion did synergize to activate caspases-9 and −7 (Fig. 4b, f, g). Bid levels decreased due to eIF5B-depletion and/or TRAIL treatment, without the concomitant appearance of tBid (Fig. 4b, h). We also failed to detect any tBID with a tBid-specific antibody (Fig. 4b). This might indicate that tBID is not formed at detectable levels in this cell line under these conditions. Interestingly, TNF-α caused no activation of caspases-8 or −3, and no further cleavage of PARP, than eIF5B-silencing alone (Figure S2A). This might be explained by the enhanced levels of the caspase-8/10 inhibitor, c-FLIP_S_, in TNF-α-treated cells (Figure S2A) and is consistent with the observation that eIF5B caused no significant sensitization to TNF-α (Fig. 1a). Taken together, these data suggest that eIF5B promotes resistance of U343 cells to TRAIL-induced apoptosis specifically by a pathway involving caspases-8, −9, and −7.

### eIF5B positively regulates antiapoptotic proteins, p21, and Nrf2

**Fig. 5:**
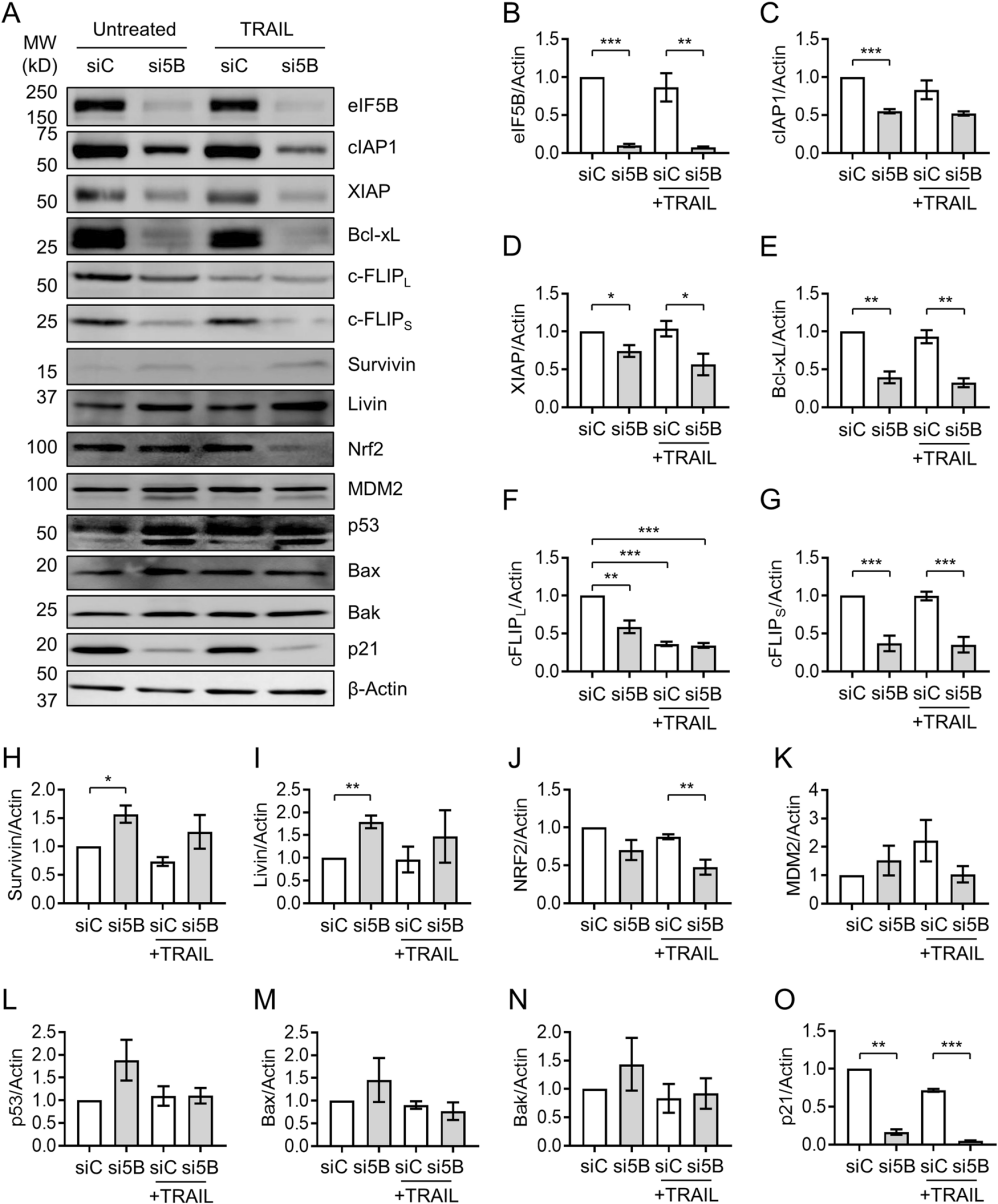
Depletion of eIF5B leads to decreased levels of antiapoptotic proteins, p21, and Nrf2. Control or eIF5B-depleted cells were treated, and protein harvested for immunoblotting, as in Fig. 4b (i.e., 100 ng/mL TRAIL for 4 h). **a** Representative images of immunoblots probing for eIF5B, cIAP1, XIAP, Bcl-xL, c-FLIP_L_, c-FLIP_S_, Survivin, Livin, Nrf2, MDM2, p53, Bax, Bak, p21, or β-actin (internal control). **b–o** Quantitation of eIF5B **b**, cIAP1 **c**, XIAP **d**, Bcl-xL **e**, c-FLIP_L_ **f**, c-FLIP_S_ **g**, Survivin **h**, Livin **i**, Nrf2 **j**, MDM2 **k**, p53 **l**, Bax **m**, Bak **n**, and p21 **o**, all normalized to β-actin. For p53 **l**, the top band (53 kDa) was quantified. Data are expressed as mean ± SEM for three independent biological replicates, except for Nrf2 (four biological replicates). *, *p* < 0.05; **, *p* < 0.01; ***, *p* < 0.001

To interrogate the mechanism by which eIF5B promotes evasion of apoptosis in U343 cells, we tested whether eIF5B depletion affects the levels of various antiapoptotic proteins. Depletion of eIF5B caused a significant reduction in the levels of cIAP1, XIAP, Bcl-xL, c-FLIP_L_, and c-FLIP_S_, in the presence or absence of TRAIL (Fig. 5a–g and S5A). Notably, these mRNAs possess IRESs. However, this effect was not observed for IAPs lacking IRESs: survivin, livin, and cIAP2 (Fig. 5a, h, i and S5A). This suggests that eIF5B specifically regulates those antiapoptotic proteins that can be translated by non-canonical mechanisms. Interestingly, XIAP decreased significantly only in U251N and U343 (Figure S5A)—those cell lines that display the eIF5B-depletion/TRAIL viability phenotype (Fig. 1a and S1C). SMC decreased levels of cIAP1 but not XIAP, c-FLIP_S_ or Bcl-xL, as expected^23^ (Figure S2B). Depletion of eIF5B caused a decrease in cIAP1, XIAP, c-FLIP_S_, and Bcl-xL in the presence of SMC or TRAIL + SMC (Figure S2B), consistent with the observation that eIF5B depletion further reduced viability in U343 cells treated with the combination of TRAIL + SMC (Fig. 1a).

As depletion of eIF5B decreased the levels of various IAPs, we tested whether eIF5B overexpression would lead to increased levels of these proteins. Relative to a vector control, we achieved a modest overexpression of eIF5B from a plasmid (p5B) that caused no increase in Bcl-xL or cIAP1 levels (Figure S6A). This suggests that eIF5B is self-limiting and that the levels of Bcl-xL and cIAP1 are saturating in the presence of eIF5B. In addition, the effect of eIF5B depletion on TRAIL sensitivity was epistatic with depletion of XIAP, Bcl-xL, and c-FLIP_S_ (Figure S6B), confirming that eIF5B and these proteins act in the same genetic pathway to promote TRAIL resistance. Depletion of cIAP1 did not affect TRAIL sensitivity, likely because cIAP2 has a redundant role^30^. Finally, we tested whether expression of Nrf2 or c-FLIP_S_ from plasmids could rescue the eIF5B-depletion phenotype to confirm a direct role for these proteins in the eIF5B-depletion phenotype. eIF5B silencing led to the characteristic increase in PARP cleavage (Figure S6C, D), which was reversed by expression of plasmid-borne Nrf2^31^ (Figure S6C) but not by c-FLIP_S_ (Figure S6D). Further, plasmid-borne expression of c-FLIP_S_ did not prevent the cleavage of the effector caspase-7. Together, these data confirm that eIF5B promotes evasion of apoptosis, at least in part, by regulating expression of non-canonically translated anti-apoptotic and pro-survival proteins.

**Fig. 6:**
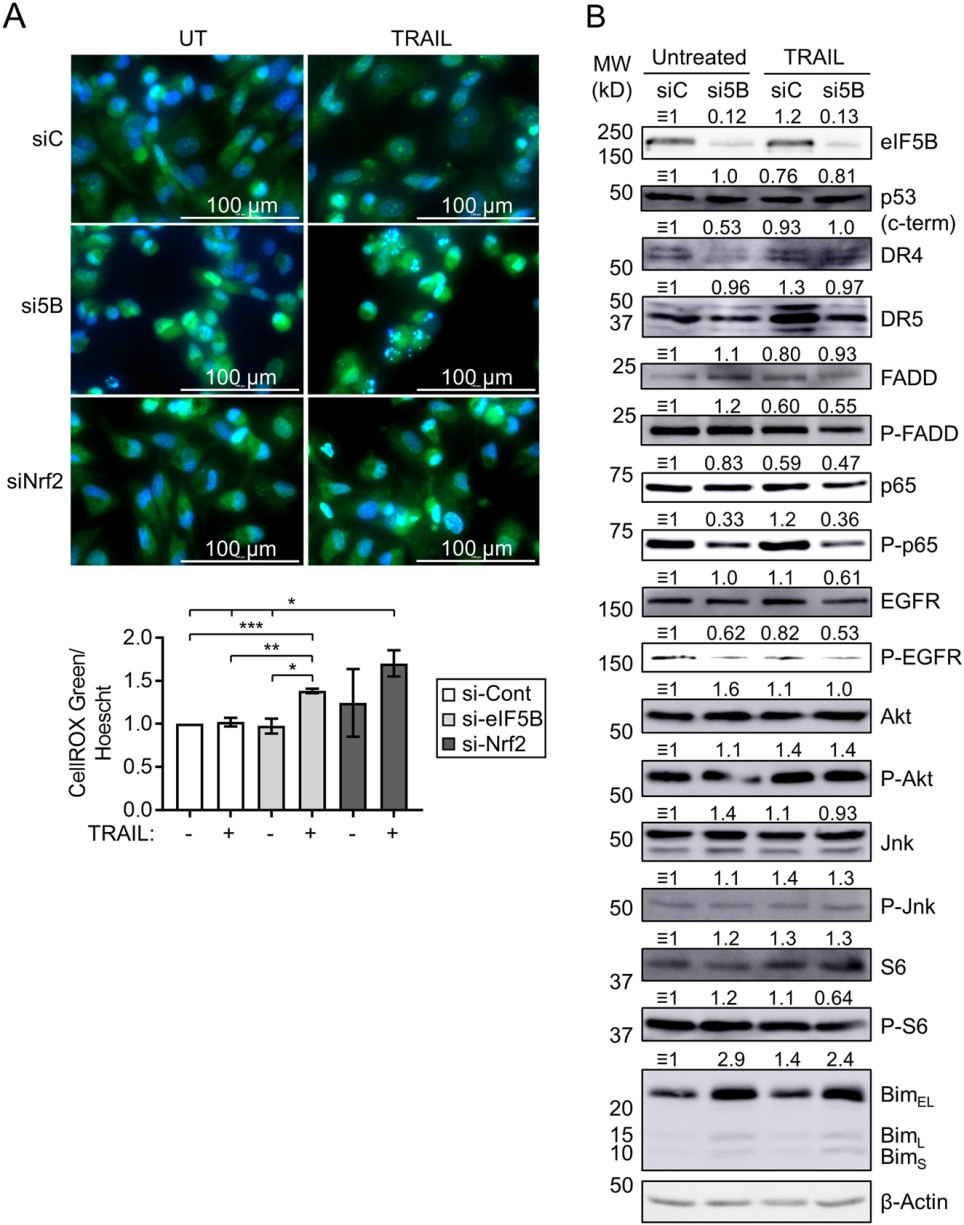
Effects of eIF5B on oxidative stress and NF-κB activation in U343. **a** Control, eIF5B-depleted, or Nrf2-depleted cells were treated with TRAIL (100 ng/mL) for 4 h before adding CellROX green reagent (5 µm; Invitrogen) and Hoechst counterstain (1 µg/mL) and incubating 30 min at 37 °C. Cells were washed with PBS before imaging at × 40 magnification in the Cytation 5 imager. Representative images are shown. The bar graph represents a quantitation of total CellROX green signal normalized to total Hoechst signal. Data are expressed as mean ± SEM for three independent biological replicates, except for siNrf2 (two biological replicates). *, *p* < 0.05; **, *p* < 0.01; ***, *p* < 0.001. **b** Immunoblots probing for p53 (C-terminus), DR4, DR5, FADD, phospho-FADD, NF-κB p65, phospho-p65, EGFR, phospho-EGFR, Akt, phospho-Akt, Jnk, phospho-Jnk, S6, phospho-S6, Bim, or β-actin. Numbers above the images represent a quantitation, normalized to the corresponding β-actin blot for the same membrane. Only one representative image is shown for β-actin

Depletion of eIF5B led to increased PARP cleavage even in the absence of TRAIL (Fig. 4E), which could be consistent with increased accumulation of ROS. Moreover, silencing eIF5B caused an increase in CellROX^TM^ green staining in the presence of TRAIL (Fig. 6a), consistent with a diminished cellular response to ROS under conditions of eIF5B depletion. This increase was similar in magnitude to that seen under conditions of Nrf2 deficiency (Fig. 6a). We also confirmed an increase in CellROX green staining in cells treated with H_2_O_2_ (Figure S7). As Nrf2, the master regulator of oxidative stress response, can be translated via an IRES^6^, we tested whether eIF5B affects the levels of Nrf2. Depletion of eIF5B caused significantly decreased levels of Nrf2 in U343 cells (Fig. 5a, j). This was the case for all but one GBM cell line tested (U87MG; Figure S5A), suggesting that eIF5B promotes cell survival by upregulating oxidative stress–response factors.

As eIF5B has been implicated in affecting the cell cycle via regulation of p21 and p27^20^, we tested whether eIF5B affects the levels of these proteins, as well as upstream regulators, in U343 cells. We did not see significant changes in levels of Mdm2, p53, or its targets^32^, Bax and Bak (Fig. 5a, k–n), although a smaller isoform of p53 was formed under these conditions. This smaller isoform is not ΔN-p53, as an antibody specific to the c-terminus of p53 (pAB421) detected uniform levels of p53 (Fig. 6b). However, levels of p21 decreased in eIF5B-depleted U343 cells (Fig. 5a,  o and Figure S5A), whereas p27 levels increased (Figure S5A), likely explaining why eIF5B-depletion had no net effect on U343 cell cycle progression (Fig. 3). Silencing p27 led to increased activation of caspase-9 (Figure S5B), indicating a pro-survival role of p27 in U343 cells—consistent with a role for p27 in promoting cytoprotective autophagy^33^. These data suggest that eIF5B plays opposing roles in regulating p21 and p27.

The pro-survival NF-κB pathway is a critical antiapoptotic mechanism^34^. In fact, a study of various glioma cell lines revealed no correlation between TRAIL sensitivity and the expression of TRAIL receptors DR4/5, but noted that TRAIL-resistant GBM lines had a high level of basal NF-κB activity^35^. Similarly, we observed a minimal effect of TRAIL on DR4/5 levels in U343 (Fig. 6b). Depletion of eIF5B led to a decrease in DR4/5 levels, as well as decreased phosphorylation of FADD in the presence of TRAIL (Fig. 6b), despite increased TRAIL sensitivity under these conditions (Fig. 1a). We thus tested whether eIF5B depletion would affect NF-κB activity. Indeed, silencing of eIF5B caused a decrease in phosphorylated p65 relative to total p65, in the presence or absence of TRAIL (Fig. 6b). This indicates decreased activation of the canonical NF-κB pathway upon eIF5B silencing. Similarly, levels of phosphorylated EGFR decreased relative to total EGFR upon silencing of eIF5B (Fig. 6b), suggesting a role for eIF5B in EGFR activation. We therefore assessed the impact of eIF5B depletion on downstream targets of EGFR. EGFR leads to phosphorylation and activation of Jnk via Ras/MAPK and Akt via PI3 kinase^36^ which, in turn, leads to phosphorylation of S6 via activated mTOR^37^. Depletion of eIF5B had no effect on the levels of total or phosphorylated Akt, Jnk, or S6 (Fig. 6b). Interestingly, total levels of Bim increased upon eIF5B depletion (Fig. 6b). Bim, a pro-apoptotic Bcl-2 family member, is regulated by the Raf-ERK pathway, wherein ERK phosphorylates Bim, leading to its ubiquitination and proteolytic degradation^38^. The enhanced levels of Bim indicate that the MAPK-Raf-ERK pathway might be inhibited by eIF5B depletion, resulting in the stabilization of Bim protein.

Collectively, the results in this section suggest that eIF5B promotes evasion of apoptosis in GBM at least partially by upregulating non-canonically translated antiapoptotic proteins (XIAP, Bcl-xL, cIAP1, and c-FLIP_s_). In addition, eIF5B promotes oxidative stress response by upregulating Nrf2, represses Bim levels, plays opposing roles in regulating p21 and p27, and promotes activation of the pro-survival NF-κB pathway.

### eIF5B enhances translation of antiapoptotic proteins, p21, and Nrf2

**Fig. 7:**
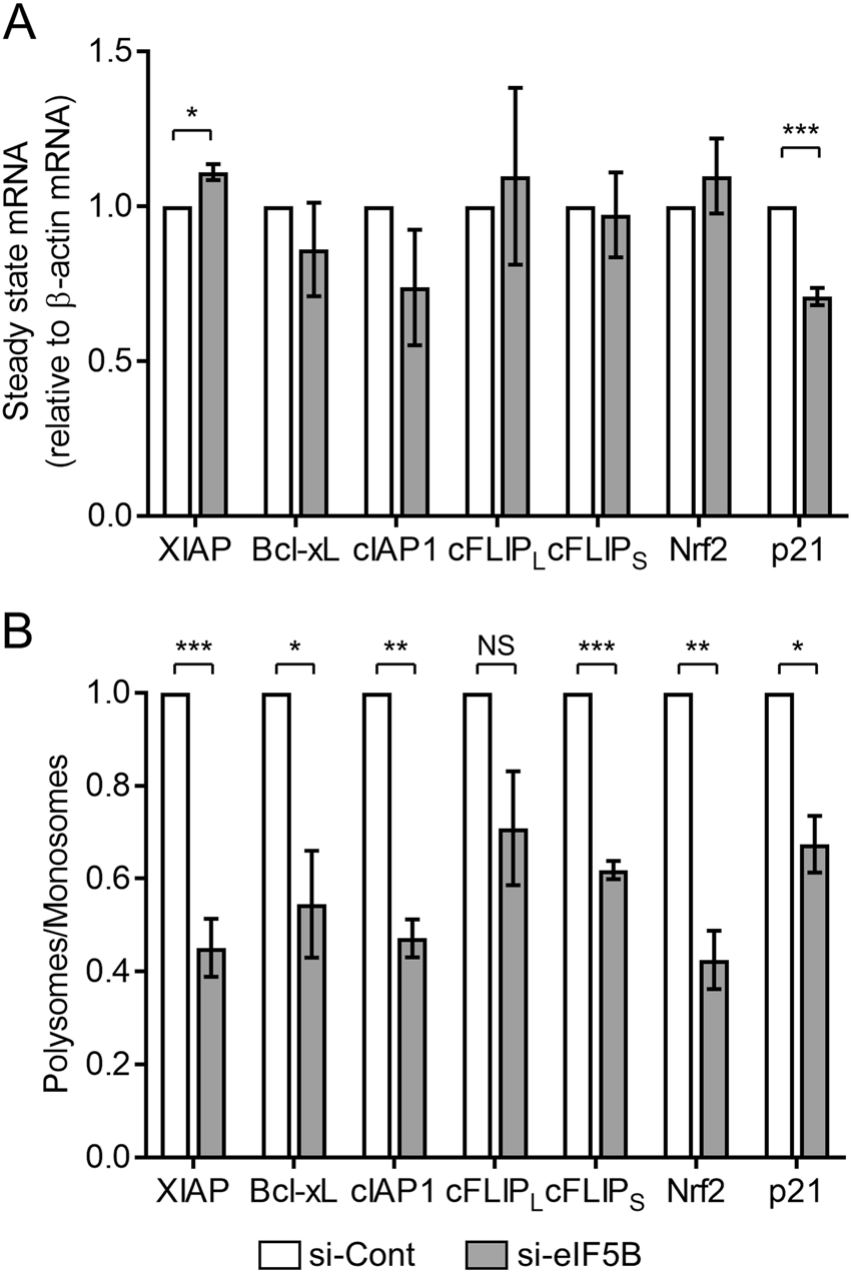
eIF5B promotes translation of XIAP, Bcl-xL, cIAP1, c-FLIP_S_, Nrf2, and p21 in U343. **a** Total RNA was isolated from control or eIF5B-depleted cells and subjected to RT-qPCR analysis to quantitate steady-state levels of cIAP1, c-FLIP_L_, c-FLIP_S_, Bcl-xL, XIAP, Nrf2, and p21 mRNAs, all normalized to β-actin mRNA. **b** Polysome profiling analysis of the aforementioned mRNAs. The proportion of each mRNA (relative to β-actin) for each fraction is shown in Figure S8. Fractions 1–3, representing monosomes, were pooled, as were fractions 4–10, representing polysomes. Data are expressed as mean ± SEM for three independent biological replicates. *, *p* < 0.05; **, *p* < 0.01; ***, *p* < 0.001

We showed in the previous section that eIF5B depletion causes a decrease in the steady-state levels of various pro-survival proteins. We next determined whether eIF5B promotes expression of these proteins at the level of translation. Steady-state levels of the mRNAs encoding XIAP, Bcl-xL, cIAP1, c-FLIP_L,_ c-FLIP_S_, and Nrf2 were not significantly affected by eIF5B depletion, whereas p21 levels decreased modestly (Fig. 7a), suggesting that eIF5B regulates these proteins post-transcriptionally. To confirm a role for eIF5B in the translation of these mRNAs, we conducted polysome profiling to determine the association of these mRNAs with translating polyribosomes versus monoribosomes. In this procedure, cell lysates are fractionated to separate monosomes from polysomes. The RNA is isolated, and RT-qPCR performed to measure the association of an mRNA of interest with each fraction. The ratio of mRNA associated with polysomes versus monosomes indicates translation efficiency, independent of mRNA steady-state levels^7,39^. The overall polysome profile of U343 cells was not drastically altered by silencing eIF5B (Figure S8A), indicating a minimal effect of eIF5B depletion on global translation. However, the proportion of XIAP, Bcl-xL, cIAP1, c-FLIP_S_, Nrf2, and p21 mRNAs associated with polysomes versus monosomes decreased in response to eIF5B depletion (Fig. 7b and S8B-H), indicating decreased translation of these mRNAs. No such trend was observed for c-FLIP_L_ (Fig. 7b and S8E). Together, the results indicate that eIF5B regulates XIAP, Bcl-xL, cIAP1, c-FLIP_S_, Nrf2, and p21 at the translational level, suggesting that eIF5B plays a direct role in regulating the translation of these mRNAs.

## Discussion

**Fig. 8:**
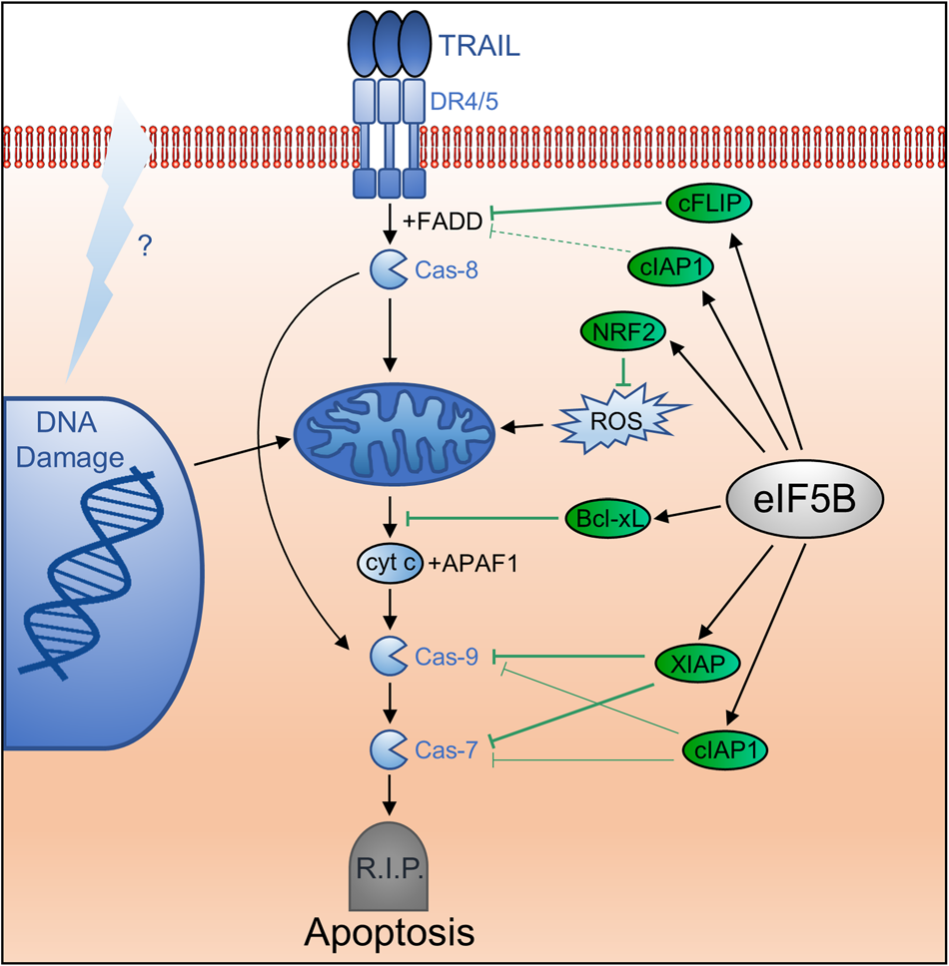
Model for regulation of apoptosis by eIF5B in U343 glioblastoma cells. eIF5B (gray oval) promotes translation of XIAP, Bcl-xL, cIAP1, c-FLIP_S_, and Nrf2 (green ovals). These, in turn, promote cell survival in the presence of TRAIL by abrogating the effects of reactive oxygen species (ROS) and/or the functions of FADD, caspase-9 (cas-9), and caspase-7 (cas-7). As XIAP, cIAP1, and Bcl-xL all act downstream of mitochondria, eIF5B might also promote resistance to intrinsic apoptosis—for instance, owing to DNA damage from chemotherapy or radiation treatment (represented by the lightning bolt)

In this work, we identify eIF5B as a pro-survival factor that regulates an alternative translation program, rendering GBM cells resistant to TRAIL-induced apoptosis (summarized in Fig. 8). Our findings are consistent with a body of work suggesting that eIF5B is involved in regulating hepatocellular carcinoma proliferation and metastasis^40^, pro-growth pathways^41^, central carbon metabolism, and hypoxia adaptation of glioblastoma^19^.

No correlation was observed between the eIF5B/TRAIL viability phenotype and standard genetic markers used to classify gliomas (p53, PTEN, and MGMT). However, U343 and U251N—those cell lines that were sensitized to SMC and/or TRAIL (Fig. 1a and S1C)—both displayed decreased phosphorylation of EGFR upon eIF5B silencing (Fig. 6b and S1C, D), suggesting a role for eIF5B in EGFR activation and a possible genetic marker for those cells that can be sensitized to apoptosis by targeting eIF5B. EGFR activates multiple signaling pathways, including the PI3K/Akt pathway, the phospholipase C/PKC signaling cascade, and the Ras/MAPK pathway^36^. Depletion of eIF5B also led to decreased activation of the NF-κB pathway (Fig. 6b). NF-κB-dependent transcription is linked to EGFR activation^42^, consistent with a positive role of eIF5B downstream of EGFR activity. Activation of NF-κB leads to enhanced expression of pro-survival and antiapoptotic genes, including XIAP, c-FLIP, and cIAP1/2^43-45^. Activation of NF-κB is hence one mechanism by which some cell lines are TRAIL-resistant (e.g., U373, U87MG; Figure S1C) despite proper expression of TRAIL receptors (DR4/5)^35^.

The effect of silencing eIF5B on TRAIL sensitivity was epistatic with the effects of silencing Bcl-xL, c-FLIP_S_, and XIAP (Figure S6), confirming that eIF5B functions in the same molecular pathway as these proteins. Silencing eIF5B caused a significant decrease in XIAP levels in the cell lines that were sensitized to TRAIL (U343 and U251N; Figure S5A), suggesting that regulation of XIAP is an important mechanism by which eIF5B promotes TRAIL resistance in these cells. However, depletion of eIF5B further sensitized U343 cells to the combination of SMC + TRAIL (Fig. 1a). As the SMC used here (TL-32711) inhibits XIAP and degrades cIAP1 and 2 via poly-ubiquitination^23^, this suggests that eIF5B depletion affects more than just these IAPs. Further, eIF5B depletion sensitized cells to SMC alone (i.e., in the absence of an obvious pro-apoptotic stimulus) (Fig. 1a), which could be explained by an increased burden of cellular ROS owing to the decreased translation of Nrf2 (Figs. 5 and 6). Increased oxidative stress would cause increased mitochondrial depolarization and hence, increased caspase activation^46^. Finally, eIF5B depletion resulted in increased levels of Bim (Fig. 6b) and decreased levels of Bcl-xL and c-FLIP_S_, which, along with XIAP, cIAP1, and Nrf2 (Fig. 5), could lead to more robust apoptosis. Indeed, eIF5B depletion was shown to enhance TRAIL-induced activation of caspases-9 and -7 (Fig. 4). Surprisingly, although eIF5B depletion led to a general disruption of the mitochondrial network in TRAIL-treated cells (Fig. 2d–f), cyt c release did not increase (Figure S4). In mice, caspase-9 can be activated by caspase-8 independently of cyt c or APAF1^47,48^. Moreover, SMAC has been shown to lead to caspase-9 activation without the concurrent release of cyt c in human multiple myeloma cells^49^. Interestingly, caspase-3 was not further activated by silencing eIF5B in TRAIL-treated U343 cells (Fig. 4b, d), indicating a degree of specificity. TRAIL was previously shown to induce caspase-9/7-dependent (but caspase-3-independent) apoptosis in a caspase-8/10-deficient neuroblastoma cell line^50^, providing a precedent for a specialized function of caspase-7.

As IAPs are key regulators of many biological processes^51^, novel therapies have attempted to target their regulation and production^10^. Targeting IAPs using small molecules sensitizes glioblastoma multiforme (GBM) cells to apoptosis^52–54^. For instance, SMCs have been used to inhibit IAPs in combination with TRAIL^22^ and oncolytic viruses^55^. Nrf2 is also emerging as a therapeutic target for glioma treatment^56^. Of the panel of proteins investigated in this work, it is notable that only those with bona fide (XIAP, Bcl-xL, cIAP1, Nrf2) or putative (c-FLIP_S_) IRESs were translationally up-regulated by eIF5B^4,6–9^. An exception is p21, which has no known IRES. However, p21 does possess an alternative non-canonical translational element—a uORF^57^. Unlike c-FLIP_S_, c-FLIP_L_ encodes no putative IRES, and was not translationally regulated by eIF5B (Fig. 7). As eIF5B was previously confirmed to play an important role in the IRES-dependent translation of XIAP^7^, we suggest that cap-independent translation mechanisms represent a common feature of those mRNAs that are at least conditionally dependent on eIF5B for efficient translation. Given that eIF5B positively regulates such a wide variety of pro-survival proteins and is largely dispensable under normal growth conditions (Figure S1A, B)^19^, we suggest that eIF5B represents a novel target to sensitize difficult-to-treat cancers to pro-apoptotic stimuli. Importantly, eIF5B-silencing did not increase TRAIL sensitivity in WI-38 and HEK293T (Fig. 1 and Figure S1). HEK293 cells express low levels of DR4/5 but can be sensitized to TRAIL by compounds that increase DR4/5 expression^58^. Similarly, WI-38 cells display TRAIL resistance, decreased caspase-8 levels, and incomplete caspase-8 activation by TRAIL, relative to cancerous cells^24^. This is consistent with our data, which shows that the eIF5B-depletion/TRAIL-sensitization phenotype requires caspase-8 activity (Fig. 4a). Taken together, the data suggest that those cancers which rely most heavily on the cap-independent translation of pro-survival proteins will be most affected by targeting eIF5B.

## Materials and methods

### Cell culture and reagents

All cell lines were propagated in Dulbecco’s high modified Eagle’s medium (DMEM; HyClone) with 4 mm l-glutamine, 4500 mg/L glucose, and 1 mm sodium pyruvate, supplemented with 10% fetal bovine serum (Gibco) and 1% penicillin–streptomycin (Gibco). Cells were incubated at 37 °C in a humidified 5% CO_2_ incubator. Cell lines were routinely tested for mycoplasma contamination with a PCR mycoplasma detection kit (ABM). The identity of U343 was verified by STR analysis. Reverse transfections were carried out using Lipofectamine RNAiMAX (Invitrogen) according to manufacturer’s instructions. Non-specific control siRNA (siC) was obtained from Qiagen. Stealth RNAi^TM^ siRNAs targeting eIF5B (HSS114469/70/71), XIAP (HSS100564/65/66), Bcl-xL (HSS141361/62/63), and cIAP1 (HSS100558/179382/179383) were obtained from Invitrogen. An siGenome SMARTpool of siRNAs targeting eIF3F (M-019535-02-0005) and a custom SMARTpool targeting c-FLIP_S_ (GETRA-000004/6/8) were obtained from Dharmacon. TL-32711 SMC was purchased from Active Biochem, TRAIL from Millipore-Sigma, and recombinant TNF-α from Bio Basic, Inc. z-VAD-fmk was obtained from Promega. Necrostatin-1 and calpain inhibitor III were purchased from Millipore-Sigma. z-LEHD-fmk, z-IETD-fmk, and 3-methyladenine were obtained from R&D systems.

### In vitro viability assay

Cell lines were seeded at 10,000 cells/well and reverse transfected in 96-well plates. After 24 h of incubation, cells were treated with a vehicle control (DMSO), TL-32711 SMC, TRAIL, or TNF-α. Where indicated, cells were pre-treated for 2 h with z-VAD-fmk, Necrostatin-1, 3-methyladenine (3MA), Calpain Inhibitor III, z-LEHD-fmk, or z-IETD-fmk before adding the compounds. After a further 72 h, cell viability was determined by alamarBlue assay (Resazurin sodium salt; Sigma-Aldrich). Fluorescence (excitation, 560 nm; emission, 590 nm) was measured in a Cytation 5 plate imager (BioTek) and data were normalized to vehicle treatment.

### Western blotting

Cells were seeded at 300,000 cells/well and reverse transfected in six-well plates. After 92 h of incubation, TRAIL, TNF-α, or SMC was added. After a further 4 h, cells were harvested in radioimmunoprecipitation assay lysis buffer supplemented with protease inhibitors. Equal amounts of soluble protein (typically 20 µg per well) were resolved by sodium dodecyl sulfate polyacrylamide gel electrophoresis and transferred onto nitrocellulose membranes (GE healthcare). Individual proteins were detected by immunoblotting with the antibodies listed in Table S2. Primary antibodies were detected with anti-rabbit-HRP conjugate (Abcam) in an AI600 imager (GE) and densitometry performed using the AI600 analysis software.

### Microscopy and immunocytochemistry

U343 cells were seeded at 10,000 cells/well and reverse transfected in 96-well plates. After 92 h of incubation, cells were treated with a vehicle control (DMEM) or TRAIL (100 ng/mL) for a further 4 h. Cells were rinsed with phosphate-buffered saline (PBS) before adding 1× annexin binding buffer containing a general DNA stain (1 µg/mL Hoechst 33342; Thermo Scientific), as well as Annexin V-FITC from the FITC Annexin V/Dead Cell kit (Invitrogen) according to manufacturer’s protocols. Finally, cells were imaged at 20× magnification in a Cytation 5 plate imager. For fluorescence microscopy, cells were imaged using a DAPI filter or a GFP filter to analyze Hoechst-stained nuclear DNA or Annexin V-FITC-positive cells, respectively. The percent of Hoechst-stained nuclei per field-of-view demonstrating fragmentation were quantified, as were the percent of annexin-positive cells. Quantitation was performed with the onboard Cytation 5 analysis software. CellROX green^TM^ was used according to manufacturer’s protocols. Alternatively, the cells were fixed in 4% formaldehyde for 15 min at room temperature and analyzed by immunocytochemistry using an Immunofluorescence solutions kit (Cell Signaling Technology) according to manufacturer’s instructions. Cytochrome c was detected with a mouse monoclonal antibody (1:300; Cell Signaling Technology #12963) and an Alexa Fluor 488-conjugated secondary antibody (1:300; Cell Signaling Technology #4408). Cells were counterstained with 1 µg/mL Hoechst 33342 and imaged by confocal microscopy at 40× magnification with z-stacking.

### Flow cytometry

U343 cells were seeded at 300,000 cells/well and reverse transfected in six-well dishes with control or eIF5B-specific siRNAs. After 24 h, TRAIL or a vehicle was added. After a further 72 h, the cells were harvested by trypsinization, rinsed with PBS, and fixed with 70% ice-cold ethanol. Fixed cells were treated with RNase A (0.625 mg/mL) before staining with 50 µg/mL Propidium Iodide at 37 °C for 30 min and determining cellular DNA content on a FACSCanto II flow cytometer (Becton-Dickinson).

### Polysome profiling and RT-qPCR

U343 cells were seeded at 1.5 million cells/plate and reverse transfected in four 10-cm Petri plates per condition. After 96 h, the control or eIF5B-depleted cells were pooled, lysed in RNA lysis buffer, and subjected to polysome profiling as previously described^39^. Gradients were fractionated using a BR-188 density gradient fractionation system. RNA was isolated essentially as described^39^ except that proteinase K treatment was replaced by incubation with 1% sodium dodecyl sulfate at 65 °C for 1 min, and hot acid phenol:chloroform (5:1; Ambion) was used to extract the RNA. After ethanol precipitating the RNA, cDNA was generated from equal volumes of RNA using the qScript cDNA synthesis kit (Quanta Biosciences). Quantitative PCR was performed in a CFX-96 real-time thermocycler (Bio-Rad) with PerfeCTa SYBR Green SuperMix (Quanta Biosciences) according to manufacturer’s instructions. Primers are detailed in Table S2. Negative controls without template DNA were run in triplicate. Each reaction was run in triplicate with the following cycle conditions: 1 cycle at 95 °C for 3 min followed by 45 cycles of 95 °C for 15 s, the annealing temperature indicated in Table S2 for 35 s, and 72 °C for 1 min. A melting curve step was added to check the purity of the PCR product. This step consisted of a ramp of the temperature from 65 to 95 °C at an increment of 0.5 °C and a hold for 5 s at each step. Expression levels were determined using the standard curve method.

### Statistical analyses

Unless otherwise specified, all quantitative data represent the mean ± standard error on the mean for at least three independent biological replicates. Statistical significance was determined by an unpaired, two-tailed *t* test without assuming equal variance. The significance level was set at a *p* value of 0.05. Data were analyzed using GraphPad Prism, version 7.

## Supplementary information

Supplemental Material
