# Supplementary material for: Eukaryotic initiation factor 5B (eIF5B) provides a critical cell survival switch to glioblastoma cells via regulation of apoptosis
Source: Cell Death Dis. 2019 Jan 22;10(2):57. doi: 10.1038/s41419-018-1283-5 (PMC6342974; doi:10.1038/s41419-018-1283-5)
Supplement: Supplementary file 1 — Supplemental Material [file 41419_2018_1283_MOESM1_ESM.pdf]

Supplemental Materials and Methods

Fractionation of mitochondrial versus cytosolic proteins

U343 cells were seeded at 1.5 million cells per plate and reverse transfected in two 10-cm Petri plates per condition. After 96 hours, the control or eIF5B-depleted cells were pooled, lysed in 1.5 mL of mitochondrial isolation buffer (250 mM sucrose, 10 mM HEPES, 1 mM EGTA, 0.5 mM PMSF, pH 7.4) by Dounce homogenization, and fractionated by differential centrifugation as previously described[1]. The mitochondrial pellets were resuspended in 50 µL of RIPA lysis buffer supplemented with protease inhibitors. Western blotting was performed as described in the main text.

Table S1. Genetic markers in glioblastoma lines used in this study.

Cell line	p53	PTEN	MGMT	DR4/5	EGFR
U343	WT[2]	Mut[3]	Neg[4]	Pos[5]	WT[3]
U251N	Mut[2]	Mut[6]	Neg[7]	Pos[8]	WT[9]
A172	WT[10]	Mut[11]	Neg[12]	Pos[13]	Active mut[14]
U87MG	WT[2]	Mut[3]	Pos[4]	Pos[8]	WT[15]
U373	Mut[16]	Mut[3]	Neg[7]	Pos[17]	WT[3]

Table S2. qPCR Primers and antibodies used in this study.

Target	Primer	Sequence (5' to 3')	Annealing Temp (°C)	Reference or source
XIAP	-	QuantiTect primer assay, QT00042854	55	Qiagen
Bcl-xL	-	QuantiTect primer assay, QT00236712	55	
Nrf2	-	QuantiTect primer assay, QT00027384	55	
p21	-	QuantiTect primer assay, QT00062090	55	
cIAP1	Fw<br>Rv	TCTGGAGATGATCCATGGGTAGA<br>TGGCCTTTCATTCGTATCAAGA	51	[18]
c-FLIP$_S$	Fw<br>Rv-S	GATGTTGCTATAGATGTGGTTC<br>ATTTCCAAGAATTTTCAGATCAGGA	47	[19]
c-FLIP$_L$	Rv-L	CCTGAATGGATTTCTTCACTGG	48	
β-actin	Fw<br>Rv	TCACCCACACTGTGCCCATCTACGA<br>TGAGGTAGTCAGTCAGGTCCC	55	[20]

Company	Antibody Target	Catalogue number
Abcam	Secondary: Goat anti-rabbit-HRP conjugate	ab97051
ProteinTech	eIF5B	13527-1-AP
Bio-Rad	β-actin (hFAB Rhodamine)	12004163
Abcam	cIAP1	ab108361
	cIAP2	ab137393
	eIF1A	ab177939
	Mdm2	ab178938
	Nrf2	ab71890
	tBid	ab10640
SantaCruz Biotech.	p27	SC-521
Calbiochem	p53 (C-terminus)	pAB421
Thermo Fisher	Phospho PERK	PA5-40294
Cell Signalling Tech.	Akt	9272
	Phospho Akt	9271
	Bak	12105
	Bax	2772
	Bcl-xL	2762
	Bid	2002
	Bim	2933

	Cas-3	9662
	Cas-7	9492
	Cas-8	9746
	Cas-9	9502
	COX IV	4844
	Cytochrome c	11940
	DR4	42533
	DR5	3696
	EGFR	2232
	Phospho EGFR	3777
	FADD	2782
	Phospho FADD	2781
	c-FLIP	56343
	JNK	9252
	Phospho JNK	9251
	Livin	5471
	PARP (total)	9542
	PARP (cleaved)	9541
	p21	2947
	p53 (total)	2527
	PERK	3192
	p65	4764
	Phospho p65	3033
	Survivin	2808
	S6	2317
	Phospho S6	5364
	XIAP	2045

Supplemental References

1. Jin Hyun Jun JK, Kyung Tae Kim and Ho Joong Sung. Application of an Efficient Method for Isolation of Mitochondria from Biological Samples. *Biomedical Science Letters* 2014, **20**(3)**:** 180-184.

2. Asai A, Miyagi Y, Sugiyama A, Gamanuma M, Hong SH, Takamoto S*, et al.* Negative effects of wild-type p53 and s-Myc on cellular growth and tumorigenicity of glioma cells. Implication of the tumor suppressor genes for gene therapy. *Journal of neuro-oncology* 1994, **19**(3)**:** 259-268.

3. Sauvageot CM, Weatherbee JL, Kesari S, Winters SE, Barnes J, Dellagatta J*, et al.* Efficacy of the HSP90 inhibitor 17-AAG in human glioma cell lines and tumorigenic glioma stem cells. *Neuro-oncology* 2009, **11**(2)**:** 109-121.

4. Voss V, Senft C, Lang V, Ronellenfitsch MW, Steinbach JP, Seifert V*, et al.* The pan-Bcl-2 inhibitor (-)-gossypol triggers autophagic cell death in malignant glioma. *Molecular cancer research : MCR* 2010, **8**(7)**:** 1002-1016.

5. Cheung HH, Mahoney DJ, Lacasse EC, Korneluk RG. Down-regulation of c-FLIP Enhances death of cancer cells by smac mimetic compound. *Cancer Res* 2009, **69**(19)**:** 7729-7738.

6. Ren Y, Zhou X, Mei M, Yuan XB, Han L, Wang GX*, et al.* MicroRNA-21 inhibitor sensitizes human glioblastoma cells U251 (PTEN-mutant) and LN229 (PTEN-wild type) to taxol. *BMC cancer* 2010, **10:** 27.

7. Chahal M, Abdulkarim B, Xu Y, Guiot MC, Easaw JC, Stifani N*, et al.* O6-Methylguanine-DNA methyltransferase is a novel negative effector of invasion in glioblastoma multiforme. *Molecular cancer therapeutics* 2012, **11**(11)**:** 2440-2450.

8. Kang YJ, Kim IY, Kim EH, Yoon MJ, Kim SU, Kwon TK*, et al.* Paxilline enhances TRAIL-mediated apoptosis of glioma cells via modulation of c-FLIP, survivin and DR5. *Experimental & molecular medicine* 2011, **43**(1)**:** 24-34.

9. Torsvik A, Stieber D, Enger PO, Golebiewska A, Molven A, Svendsen A*, et al.* U-251 revisited: genetic drift and phenotypic consequences of long-term cultures of glioblastoma cells. *Cancer medicine* 2014, **3**(4)**:** 812-824.

10. Mittelbronn M, Platten M, Zeiner P, Dombrowski Y, Frank B, Zachskorn C*, et al.* Macrophage migration inhibitory factor (MIF) expression in human malignant gliomas contributes to immune escape and tumour progression. *Acta neuropathologica* 2011, **122**(3)**:** 353-365.

11. Zhang R, Banik NL, Ray SK. Differential sensitivity of human glioblastoma LN18 (PTEN-positive) and A172 (PTEN-negative) cells to Taxol for apoptosis. *Brain research* 2008, **1239:** 216-225.

12. Gaspar N, Marshall L, Perryman L, Bax DA, Little SE, Viana-Pereira M*, et al.* MGMT-independent temozolomide resistance in pediatric glioblastoma cells associated with a PI3-kinase-mediated HOX/stem cell gene signature. *Cancer research* 2010, **70**(22)**:** 9243-9252.

13. van Roosmalen IAM, Reis CR, Setroikromo R, Yuvaraj S, Joseph JV, Tepper PG*, et al.* The ER stress inducer DMC enhances TRAIL-induced apoptosis in glioblastoma. *SpringerPlus* 2014, **3:** 495.

14. Fenstermaker RA, Ciesielski MJ. EGFR Intron Recombination in Human Gliomas: Inappropriate Diversion of V(D)J Recombination? *Current genomics* 2007, **8**(3)**:** 163-170.

15. Fontana AO, Piffaretti D, Marchi F, Burgio F, Faia-Torres AB, Paganetti P*, et al.* Epithelial growth factor receptor expression influences 5-ALA induced glioblastoma fluorescence. *Journal of neuro-oncology* 2017, **133**(3)**:** 497-507.

16. Gomez-Manzano C, Fueyo J, Kyritsis AP, McDonnell TJ, Steck PA, Levin VA*, et al.* Characterization of p53 and p21 functional interactions in glioma cells en route to apoptosis. *Journal of the National Cancer Institute* 1997, **89**(14)**:** 1036-1044.

17. Hetschko H, Voss V, Horn S, Seifert V, Prehn JH, Kogel D. Pharmacological inhibition of Bcl-2 family members reactivates TRAIL-induced apoptosis in malignant glioma. *Journal of neuro-oncology* 2008, **86**(3)**:** 265-272.

18. Graber TE, Baird SD, Kao PN, Mathews MB, Holcik M. NF45 functions as an IRES trans-acting factor that is required for translation of cIAP1 during the unfolded protein response. *Cell Death Differ* 2010, **17**(4)**:** 719-729.

19. Ueffing N, Singh KK, Christians A, Thorns C, Feller AC, Nagl F*, et al.* A single nucleotide polymorphism determines protein isoform production of the human c-FLIP protein. *Blood* 2009, **114**(3)**:** 572-579.

20. Khan D, Katoch A, Das A, Sharathchandra A, Lal R, Roy P*, et al.* Reversible induction of translational isoforms of p53 in glucose deprivation. *Cell death and differentiation* 2015, **22**(7)**:** 1203-1218.

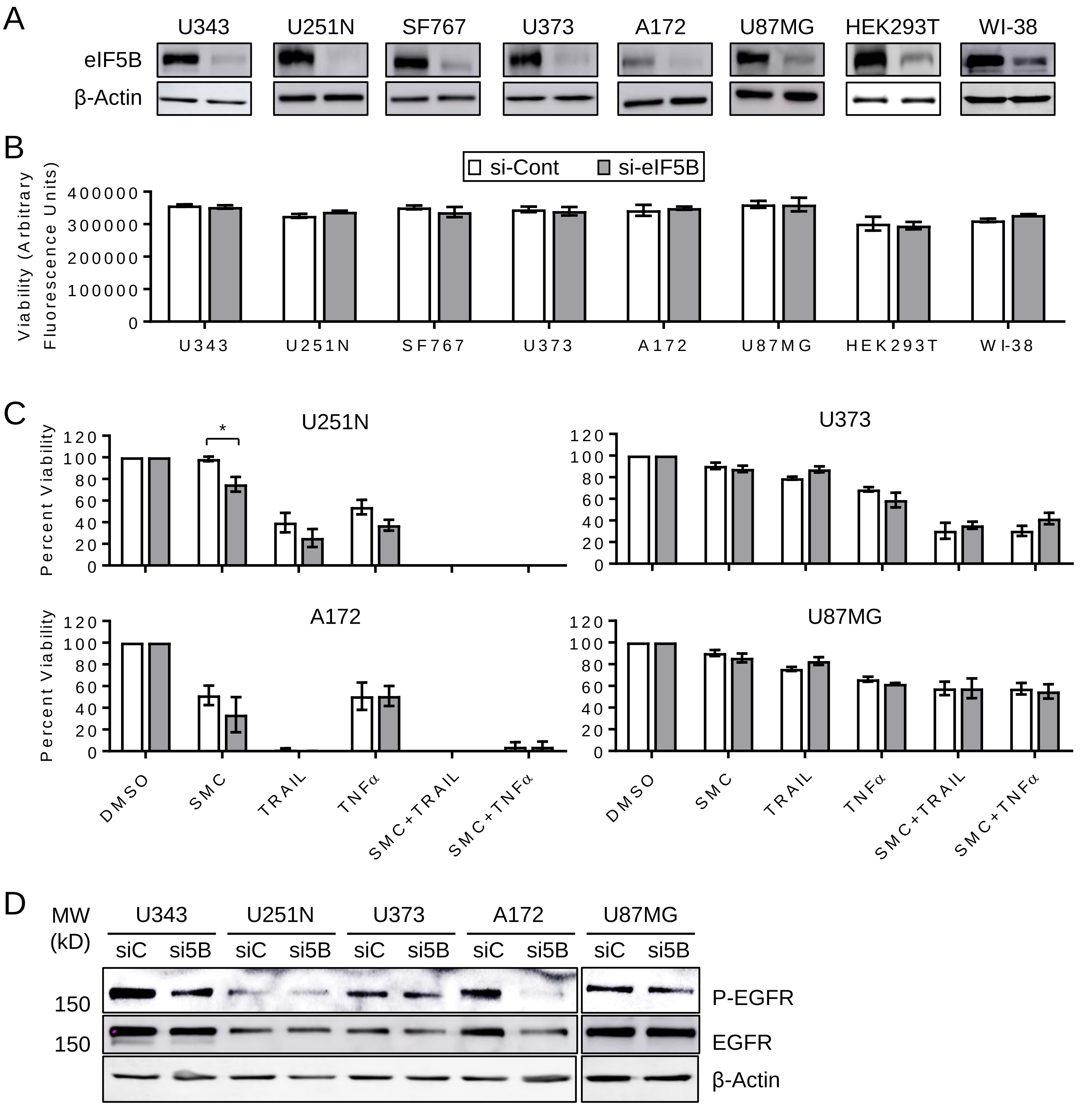

Figure S1. Effects of eIF5B depletion on various cell lines.
(A) Immunoblots confirming eIF5B silencing in various cell lines. **(B)** Depletion of eIF5B has little effect on alamarBlue activity in the tested cell lines. **(C)** Various cell lines were treated as in Figure 1. Data are expressed as mean ± SEM for three independent biological replicates. *, $p < 0.05$. **(D)** Effects of eIF5B depletion on total or phospho-EGFR (Tyr1068) in various cell lines.

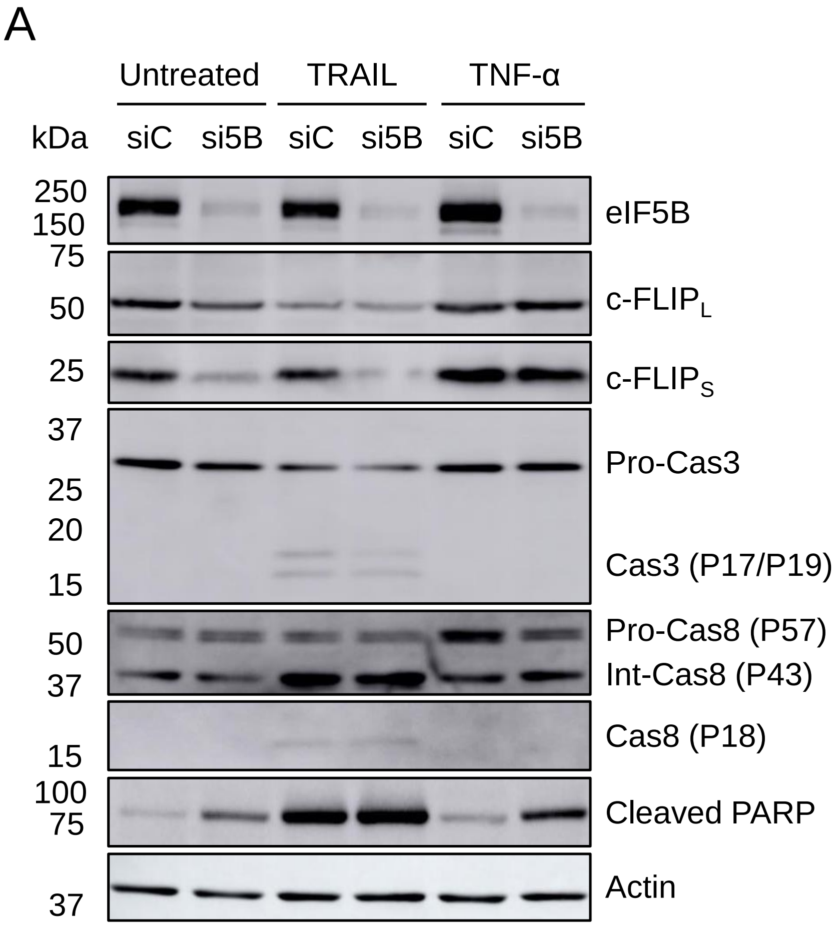

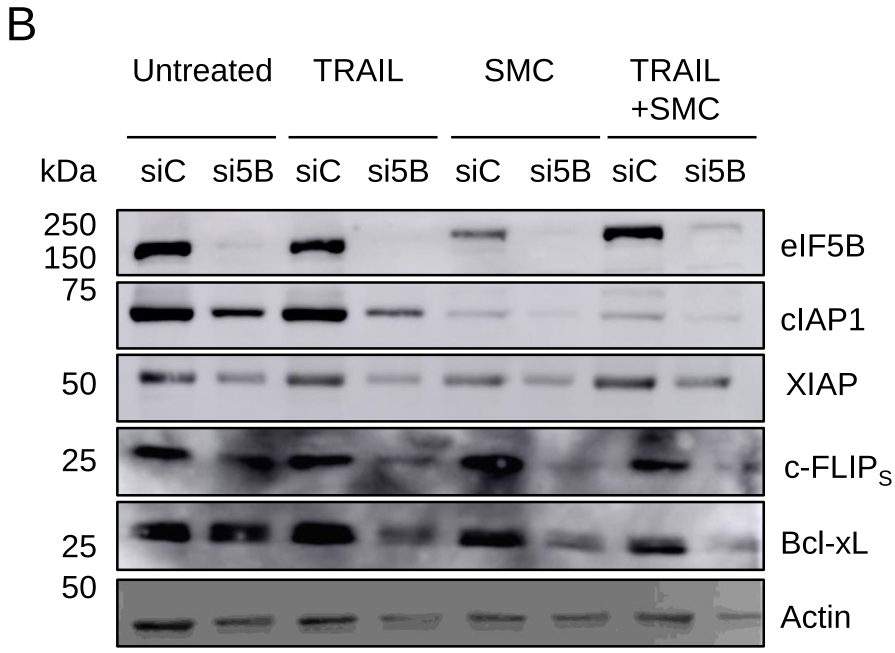

Figure S2. Effects of eIF5B depletion on the levels of anti-apoptotic proteins and caspase activation in TNF-α- and SMC-treated U343 cells.
(A) TNF-α prevents down-regulation of c-FLIP$_L$ levels upon eIF5B depletion and enhances c-FLIP$_S$ levels. Unlike TRAIL, TNF-α causes no activation of caspase-8 or -3 and no further cleavage of PARP than does eIF5B depletion alone. Lanes 1-4 for eIF5B, Cas-3, cFLIP$_L$, and cFLIP$_S$ are also shown in Figures 4B and 5A, respectively. Here we have shown the exact same lanes to compare them with TNFα lanes. **(B)** SMC decreases levels of cIAP1 but not XIAP, c-FLIP$_S$ or Bcl-xL. Depletion of eIF5B causes a decrease in cIAP1, XIAP, c-FLIP$_S$, and Bcl-xL in the presence of TRAIL, SMC, or TRAIL+SMC.

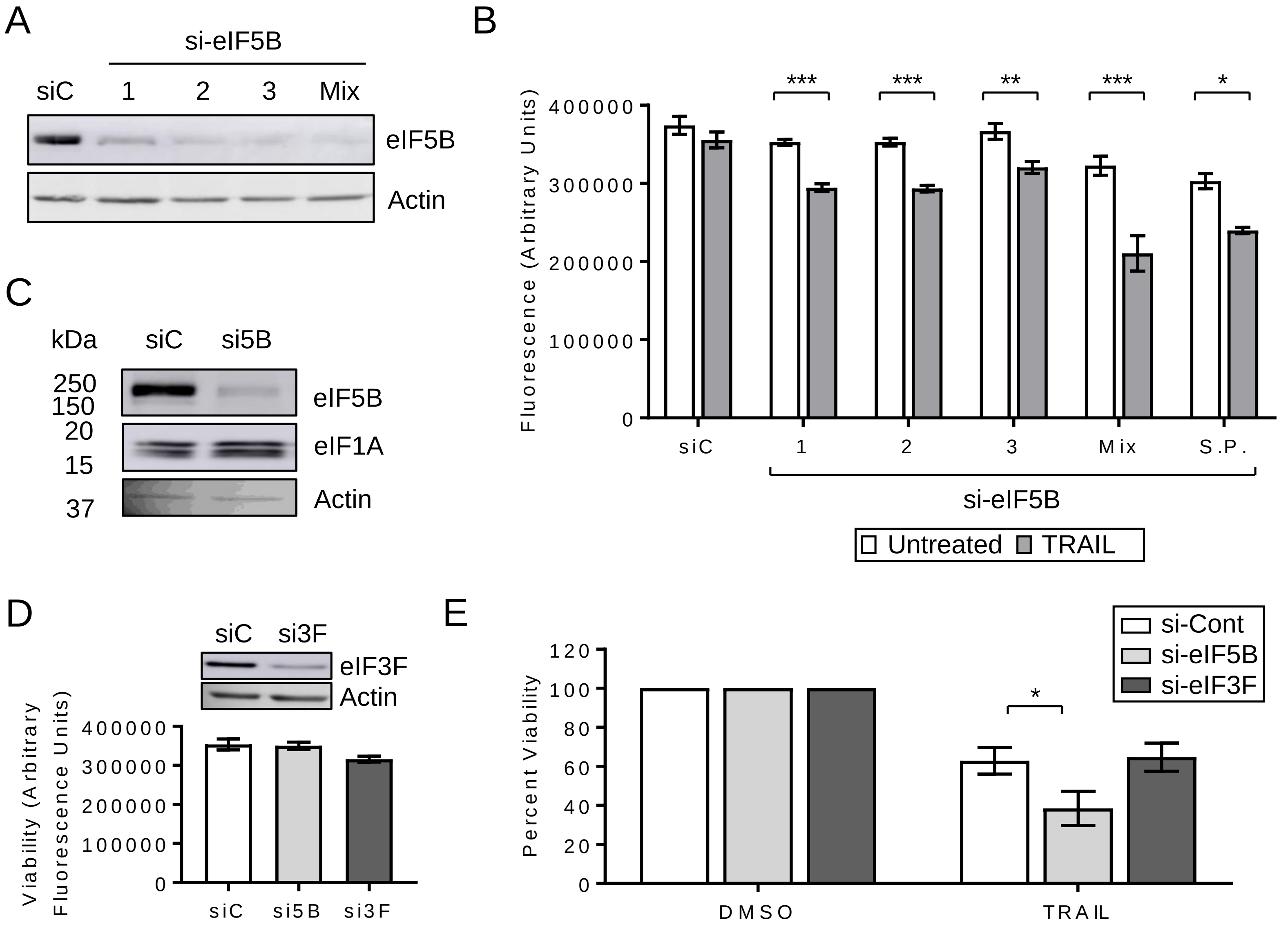

Figure S3. Controls related to Figure 1.

(A) Immunoblot confirming eIF5B silencing by the individual siRNAs or the mix of three siRNAs used throughout this study, relative to the non-specific control siRNA (siC). **(B)** Each siRNA, as well as the mix and a SMARTpool from Dharmacon (S.P.), yields increased sensitivity of U343 cells to TRAIL. **(C)** Immunoblot showing no obvious effect of eIF5B-depletion on eIF1A levels. **(D)** Like eIF5B, depletion of eIF3F alone has no significant effect on the viability of U343 cells. **(E)** Unlike eIF5B, silencing of eIF3F does not sensitize U343 cells to TRAIL. Data are expressed as mean ± SEM for 3 independent biological replicates. *, $p < 0.05$; **, $p < 0.01$; ***, $p < 0.001$.

Figure S4

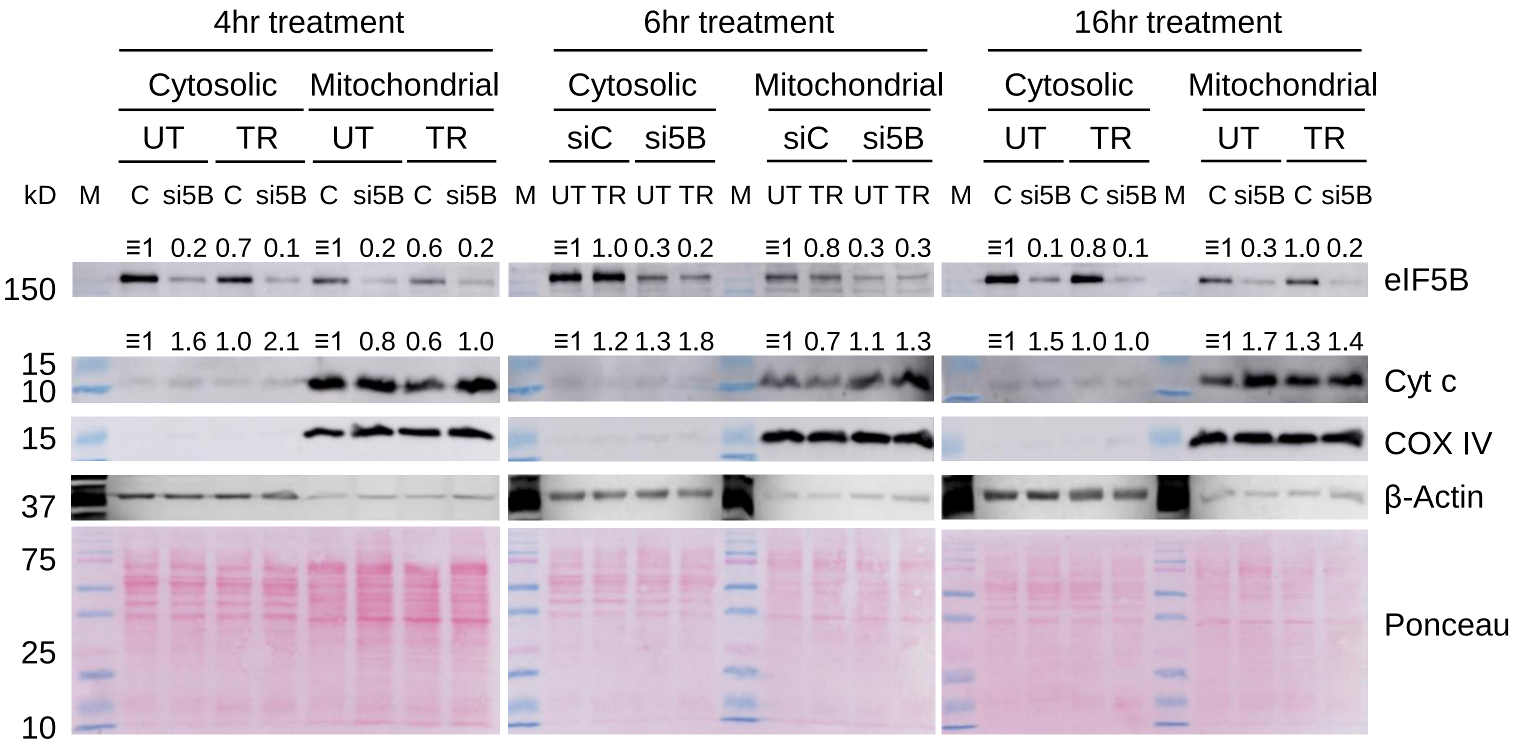

Figure S4. Immunoblots of cytosolic versus mitochondrial proteins in control versus eIF5B-depleted U343 cells.
Control or eIF5B-depleted U343 cells were harvested after a total of 96 hours of incubation; TRAIL was added to 100 ng/mL for the last 4, 6, or 16 hours of incubation, as indicated. Immunoblotting was performed for eIF5B, cytochrome c (Cyt c), β-actin (internal standard for cytosolic fractions), or cytochrome oxidase (COX IV; internal standard for mitochondrial fractions). A representative image is shown for a total protein stain of the membranes (Ponceau). The numbers above the blots represent a quantitation of eIF5B or cytochrome c, normalized to β-actin (in the case of the cytosolic fractions) or COX IV (in the case of the mitochondrial fractions).

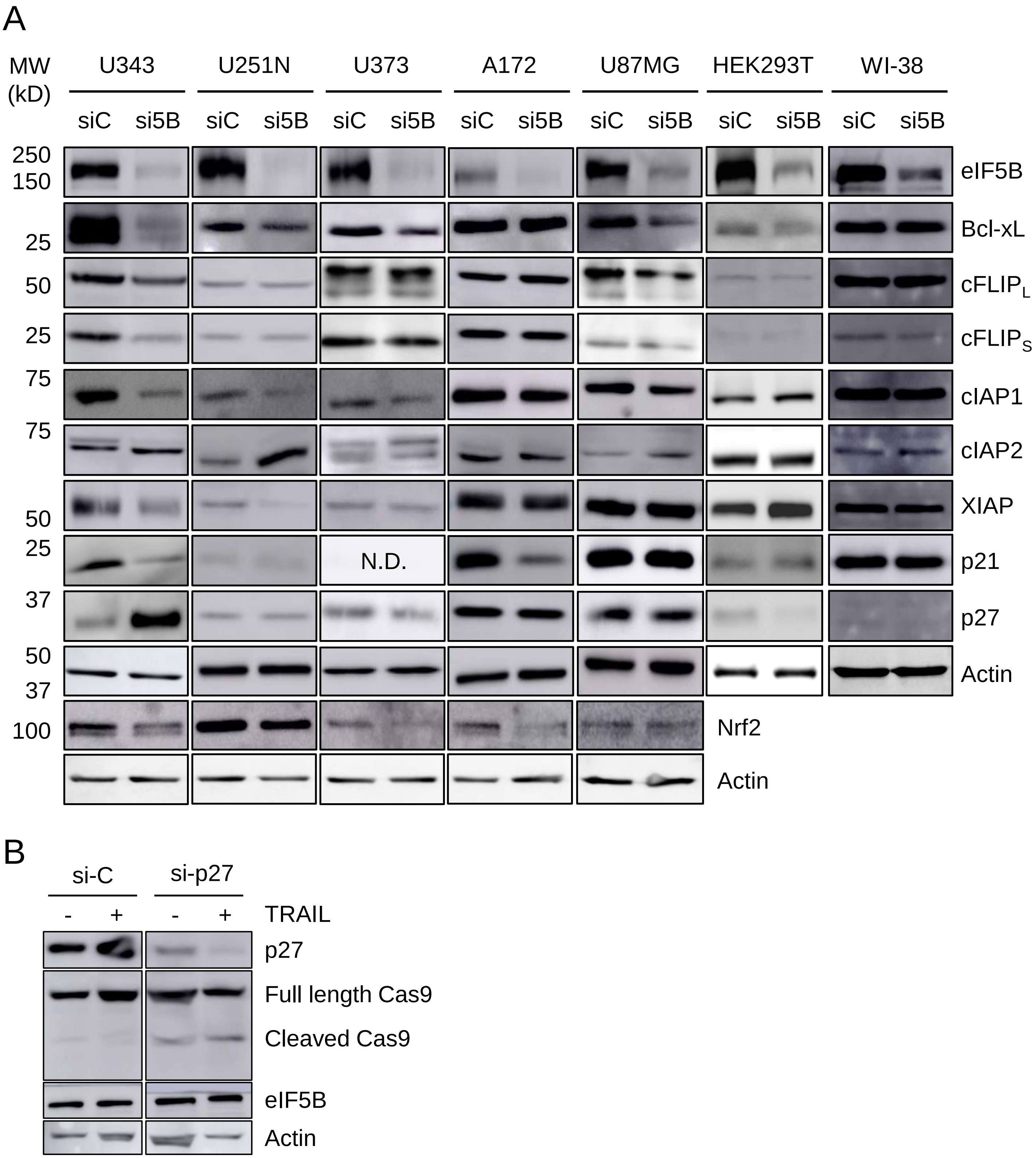

Figure S5. Effects of eIF5B on pro-survival proteins.
(A) Effects of eIF5B depletion on anti-apoptotic proteins, p21, p27, and Nrf2 in various cell lines. N.D., not detected. **(B)** Depletion of p27 leads to increased caspase-9 activation in U343 cells.

Figure S6

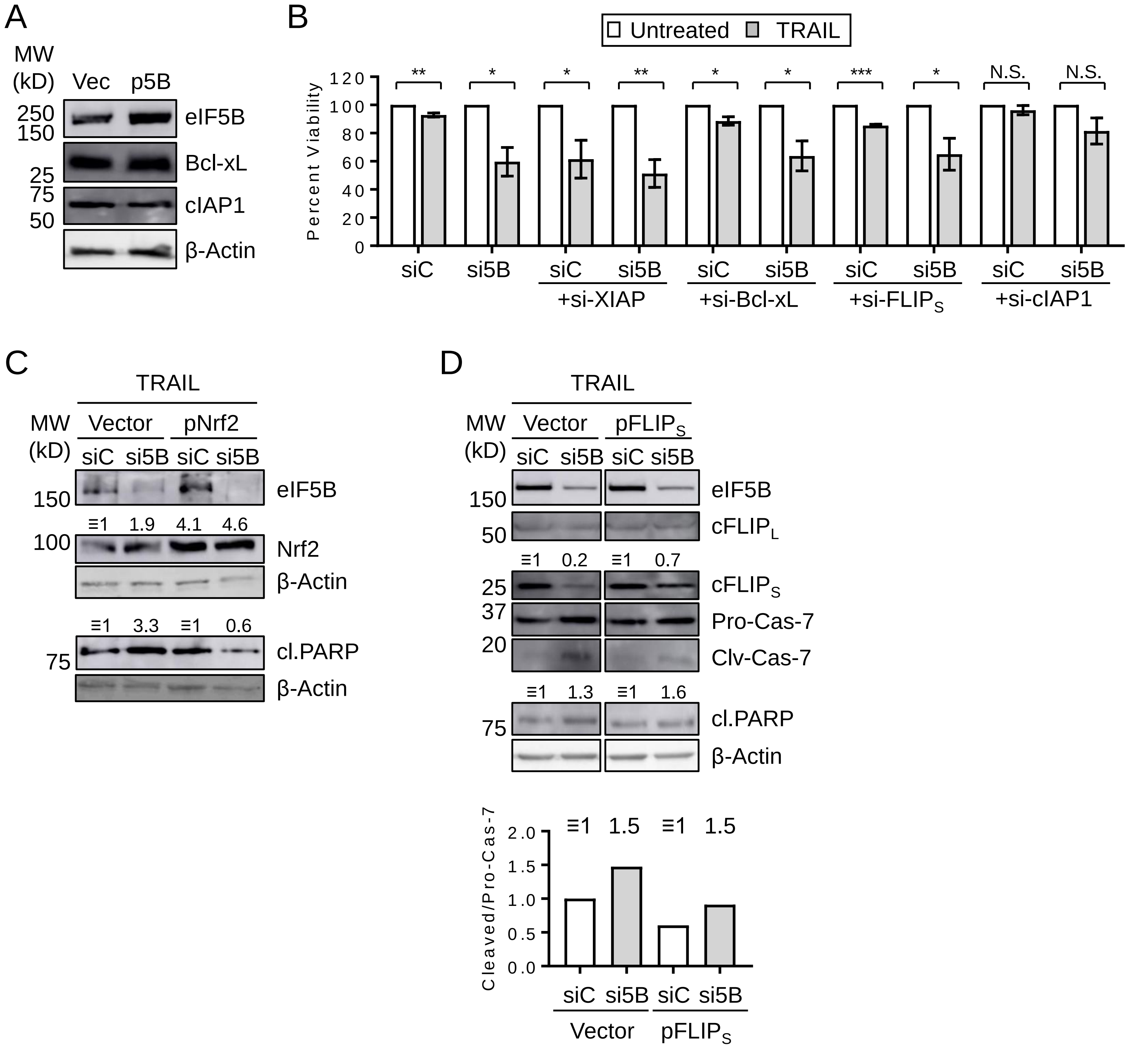

Figure S6: The effect of eIF5B depletion on TRAIL sensitivity is exerted via several IAPs and Nrf2. (A) Modest over-expression of eIF5B from a plasmid (p5B), obtained from Dharmacon (OHS6085-213579952), had little effect on Bcl-xL or cIAP1 levels. **(B)** The effect of eIF5B depletion on TRAIL sensitivity is epistatic with depletion of XIAP, Bcl-xL, and c-FLIP$_S$. U343 cells were treated essentially as in Figure 1, except that eIF5B was depleted either alone or in combination with XIAP, Bcl-xL, c-FLIP$_S$ or cIAP1. Data are expressed as mean ± SEM for 3 independent biological replicates. *, $p < 0.05$; **, $p < 0.01$; ***, $p < 0.001$. **(C)** Depletion of eIF5B causes increased PARP cleavage, which is reversed by expression of Nrf2 from a plasmid (pNrf2), obtained from AddGene (NC16 pCDNA3.1 FLAG NRF2). **(D)** Depletion of eIF5B causes increased cas-7 activation and PARP cleavage, neither of which is reversed by expression of c-FLIP$_S$ from a plasmid (pFLIP$_S$), a gift of Christian Schwerk. For eIF5B depletion coupled with Nrf2 or c-FLIP$_S$ overexpression, 420,000 U343 cells were seeded per well in a 6-well plate and reverse transfected with control or eIF5B-specific siRNAs. After 48 hours of incubation, the cells were transfected with the indicated plasmid. After a further 42 hours of incubation, the cells were treated with 100 ng/mL TRAIL for 4 hours before being harvested for western blotting.

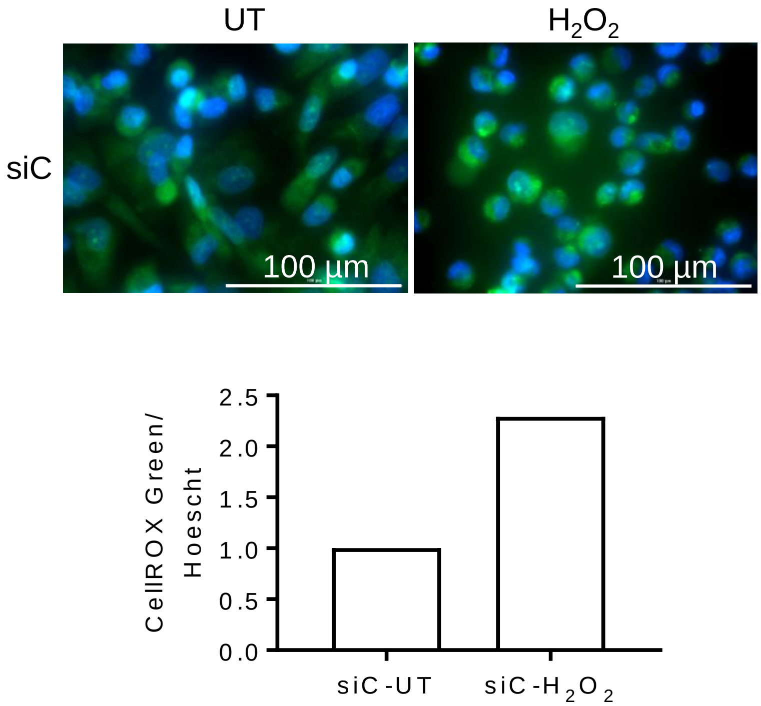

Figure S7. CellROX green staining of untreated and H_2O_2-treated U343 cells (related to Figure 6E). U43 cells were treated with siC as in Figure 6A. Instead of TRAIL, cells were treated for 4 hours with 200 µM H_2O_2 or left untreated, before staining with CellROX green and processing as in Figure 6A.

Figure S8

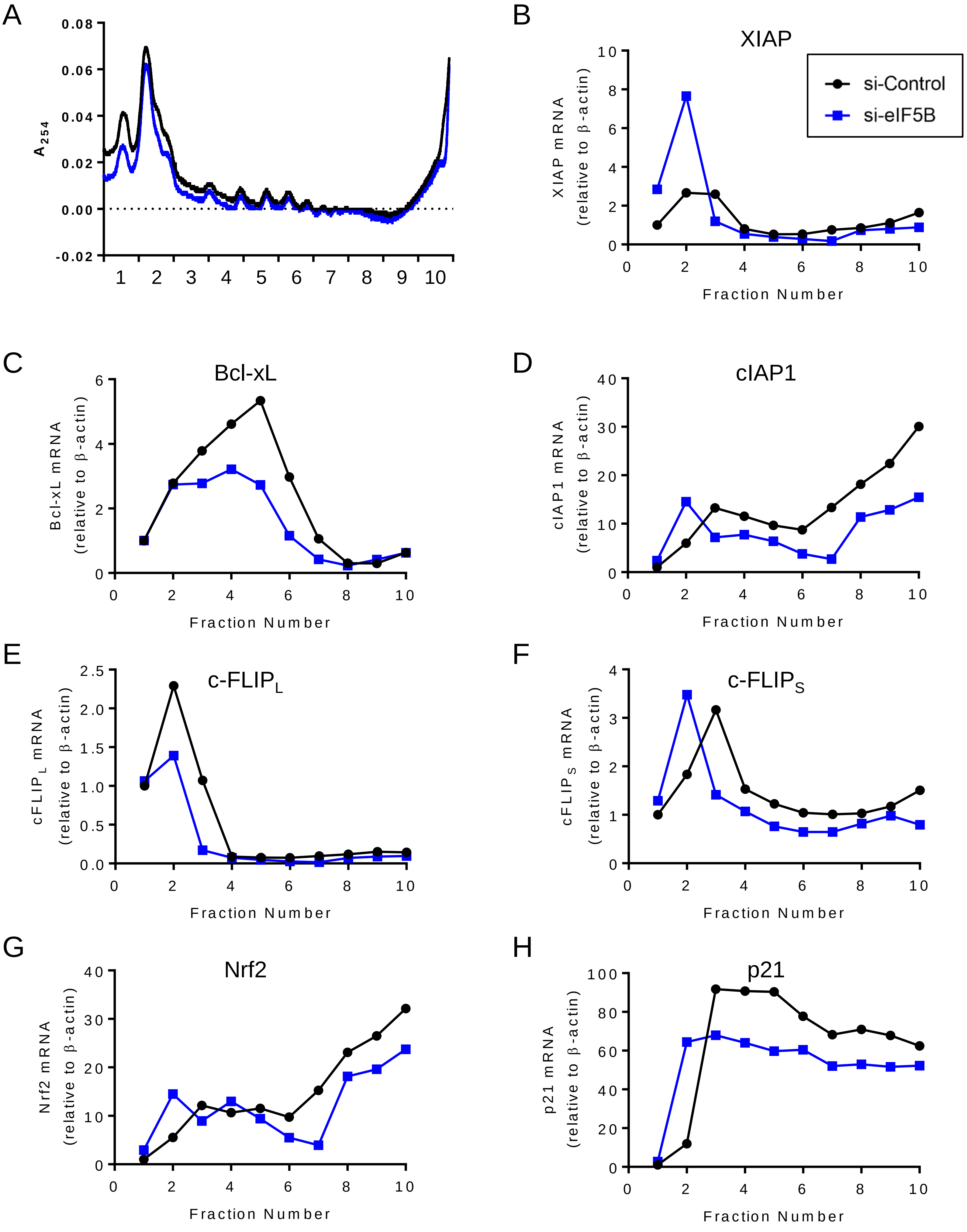
A
A_{254}
0.08
0.06
0.04
0.02
0.00
-0.02
1 2 3 4 5 6 7 8 9 10
B
XIAP
XIAP mRNA (relative to β-actin)
si-Control
si-eIF5B
Fraction Number
C
Bcl-xL
Bcl-xL mRNA (relative to β-actin)
Fraction Number
D
cIAP1
cIAP1 mRNA (relative to β-actin)
Fraction Number
E
c-FLIP$_L$
cFLIP$_L$ mRNA (relative to β-actin)
Fraction Number
F
c-FLIP$_S$
cFLIP$_S$ mRNA (relative to β-actin)
Fraction Number
G
Nrf2
Nrf2 mRNA (relative to β-actin)
Fraction Number
H
p21
p21 mRNA (relative to β-actin)
Fraction Number

Figure S8. Representative polysome profiles for control or eIF5B-depleted U343 cells (related to Figure 7).

(A) Representative polysome profiles for control and eIF5B-depleted U343 cells (black and blue curves, respectively). **(B-H)** RNA was purified from the sucrose fractions indicated in panel (A) and subjected to RT-qPCR analysis. The proportion of mRNAs encoding XIAP **(B)**, Bcl-xL **(C)**, cIAP1 **(D)**, c-FLIP$_L$ **(E)**, c-FLIP$_S$ **(F)**, Nrf2 **(G)** and p21 **(H)**, all relative to β-actin mRNA, were quantified for each fraction.
